# Transcriptome Analysis Revealed Highly Expressed Genes Encoding Secondary Metabolite Pathways and Small Cysteine-Rich Proteins in the Sclerotium of *Lignosus rhinocerotis*

**DOI:** 10.1371/journal.pone.0143549

**Published:** 2015-11-25

**Authors:** Hui-Yeng Y. Yap, Yit-Heng Chooi, Shin-Yee Fung, Szu-Ting Ng, Chon-Seng Tan, Nget-Hong Tan

**Affiliations:** 1 Department of Molecular Medicine, Faculty of Medicine, University of Malaya, Kuala Lumpur, Malaysia; 2 School of Chemistry and Biochemistry, University of Western Australia, Crawley, Western Australia, Australia; 3 Ligno Biotech Sdn. Bhd., Balakong Jaya, Selangor, Malaysia; 4 Malaysian Agricultural Research and Development Institute (MARDI), Serdang, Selangor, Malaysia; Louisiana State University Agricultural Center, UNITED STATES

## Abstract

*Lignosus rhinocerotis* (Cooke) Ryvarden (tiger milk mushroom) has long been known for its nutritional and medicinal benefits among the local communities in Southeast Asia. However, the molecular and genetic basis of its medicinal and nutraceutical properties at transcriptional level have not been investigated. In this study, the transcriptome of *L*. *rhinocerotis* sclerotium, the part with medicinal value, was analyzed using high-throughput Illumina HiSeq^TM^ platform with good sequencing quality and alignment results. A total of 3,673, 117, and 59,649 events of alternative splicing, novel transcripts, and SNP variation were found to enrich its current genome database. A large number of transcripts were expressed and involved in the processing of gene information and carbohydrate metabolism. A few highly expressed genes encoding the cysteine-rich cerato-platanin, hydrophobins, and sugar-binding lectins were identified and their possible roles in *L*. *rhinocerotis* were discussed. Genes encoding enzymes involved in the biosynthesis of glucans, six gene clusters encoding four terpene synthases and one each of non-ribosomal peptide synthetase and polyketide synthase, and 109 transcribed cytochrome P450 sequences were also identified in the transcriptome. The data from this study forms a valuable foundation for future research in the exploitation of this mushroom in pharmacological and industrial applications.

## Introduction

The polypore *Lignosus rhinocerotis* (Cooke) Ryvarden, or generally known as tiger milk mushroom, is a highly valued medicinal mushroom of the Southeast Asia. It is mainly distributed in China, Thailand, Malaysia, Indonesia, Philippines, and Papua New Guinea [1–3]. This mushroom is traditionally being used as tonic, to maintain general health, for immune enhancement, or as a treatment regime for numerous ailments including cancer, asthma, and bronchitis. It is also used to treat discomfort caused by fright, fever, coughing, vomiting, and cuts. The mushroom’s sclerotium is the main source of medicinal material. Besides being consumed in the form of decoction (usually with other herbs), several other preparation methods for medicinal purposes have been documented [4]. In the interior of state of Pahang, tiger milk mushroom was found to be administered in a betel quid. While in the state of Kelantan, where the mushroom is often given to mothers after childbirth, its sclerotium is pounded with raw rice, infused, and drunk [5, 6]. A technique mimicking cold water extraction has been described by Chan [7] where the sclerotium is grated on a hard surface such as granite plate with some water and the resulting mixture is further diluted with water before consumed.

Since its successful cultivation, researchers have been actively validating the traditional claims by various scientific approaches. Several authors have reported studies on the chemical composition, nutritive value, and health benefits of *L*. *rhinocerotis*. While some have also demonstrated the presence of antiproliferative activity in aqueous (hot and cold) or methanol pressurized liquid extracts, and hot water-soluble polysaccharide-protein complex isolated from *L*. *rhinocerotis* sclerotium against a panel of human cancer cell lines as well as various types of leukemic cells including acute promyelocytic leukemia cells (HL-60), chronic myelogenous leukemia cells (K562), and human acute monocytic leukemia cells (THP-1), through apoptosis and/or cell cycle arrest [8–11]. Some other biomedical properties of *L*. *rhinocerotis* sclerotium such as immunomodulatory, neurite outgrowth-stimulating, antinociceptive, and antimicrobial properties have also been reported [12–17].

The genome of *L*. *rhinocerotis* was reported previously by our group. It is particularly enriched with sesquiterpenoid biosynthesis genes and appears to encode the capabilities to produce 1,3-β- and 1,6-β-glucans as well as several bioactive proteins including lectins and fungal immunomodulatory proteins [18]. With the rapid advancement of high-throughput deep sequencing technologies such as RNA sequencing (RNA-seq), application of genome-wide expression profiling on this mushroom is now possible. RNA-seq or “Whole Transcriptome Shotgun Sequencing” is a rapid and cost-effective revolutionary tool that uses the capabilities of next-generation sequencing platform to generate large numbers of high-quality short reads for comprehensive transcriptomics data mining [19, 20]. In order to shed light into the genes that are expressed in the sclerotium of *L*. *rhinocerotis*, transcriptome analysis was conducted and the data generated was mapped to its genome to further enhance gene structure annotation and novel transcripts and alternative splicing predictions [21, 22]. This is the first survey on transcriptome characterization for this mushroom where assessment of transcriptome coverage, gene sequences, and functional annotation by bioinformatics analysis were included. And in view of the availability of transcriptome data, the secondary metabolism and polysaccharides biosynthesis of *L*. *rhinocerotis* were re-examined to gain better understanding. The structural and functional features of several highly expressed genes were also discussed.

## Materials and Methods

### Strain and culture condition

Freeze-dried sclerotial powder of *L*. *rhinocerotis* TM02 cultivar was a product of LiGNO™ Biotech Sdn. Bhd. (Selangor, Malaysia). A voucher specimen was deposited at Royal Botanic Gardens, Kew (London, UK) with the accession number K(M) 177812. The fungal culture was grown in sterile rice-based solid media at 28°C in the dark for three months. Samples from the developed sclerotia were harvested and freeze dried to a completely dry form. The product was authenticated by DNA fingerprinting [23].

### RNA preparation and library sequencing

Total RNA was extracted from fresh sclerotial powder using TRIzol^®^ Reagent (Invitrogen, California, USA) according to manufacturer's instructions. A sufficient amount of total RNA of not less than 10 μg with OD_260_/OD_280_ values of 1.8 to 2.2 was used. The sample was purified and treated with DNase in order to remove protein and DNA contaminants. Sample for transcriptome analysis was prepared as per Illumina manufacturer’s instructions where magnetic beads with oligo(dT) were used to isolate polyadenylated mRNA (poly(A)^+^ RNA) from the total RNA. Fragmentation buffer consisting of divalent cations was added for interrupting mRNA to short fragments of 200 to 700 nucleotides in length. These short fragments were used as templates to synthesize the first-strand cDNA using random hexamer-primer. The second-strand cDNA was synthesized using buffer, dNTPs, RNaseH, and DNA polymerase I. The products were purified and resolved with QIAquick PCR Purification Kit (Qiagen, California, USA) and EB buffer for end reparation and tailing A, respectively. Purified cDNA fragments were connected with sequencing adapters and gel electrophoresed to select suitable fragments for PCR amplification. Agilent 2100 Bioanalyzer and Applied Biosystems^®^ StepOnePlus™ Real-Time PCR System were used in quantification and qualification of the sample library for quality control. A paired-end cDNA library was constructed and sequenced using Illumina HiSeq™ 2000 at BGI-Shenzhen, China.

### Data processing

To avoid negative impact on subsequent bioinformatics analysis, raw reads were filtered by the removal of reads with adapters, unknown nucleotides larger than 10%, and low quality reads where more than half of the bases' qualities are less than five (Q ≤ 5). Base composition and quality distribution were determined as quality control. The obtained clean reads were then mapped to the genome and genes sequences of *L*. *rhinocerotis* TM02 using Short Oligonucleotide Analysis Package 2 (SOAP2) [24] where up to five bases mismatches were allowed in the alignment. The alignment data was used to calculate distribution of reads on reference genes and mapping ratio.

### Bioinformatics analysis

For alternative splicing analysis, junction sites were detected by TopHat with default parameters [25]. Junction sites which provide information on boundaries and combinations of different exons in a transcript of the same gene were used to distinguish the type of alternative splicing event including exon skipping, intron retention, alternative 5’ splice site, and alternative 3’ splice site. For novel transcripts prediction, gene models found in intergenic regions [200 base pairs (bp) away from upstream or downstream genes] were thought to be potential candidate. A member of the SOAP, SOAPsnp was used to detect single-nucleotide polymorphism (SNPs) [24]. SNPs were identified on the consensus sequence through the comparison with the reference genome of *L*. *rhinocerotis* TM02 strain [18]. Gene models were aligned to SwissProt, TrEMBL, and NCBI nr (BLASTP cut-off e-value ≤ 1e-5) and their functional classification was annotated by Gene Ontology (GO), Cluster of Orthologous Groups (COG), and Kyoto Encyclopedia of Genes and Genomes (KEGG) pathways [26–28].

### Expression annotation

Gene expression level was determined by the reads per kilobase per million reads (RPKM) method developed by Mortazavi et al. [29] to eliminate the influence of different gene length and sequencing discrepancy. For instance, the RPKM value of gene A was calculated using the formula 10^6^∙C ÷ ((N∙L)/10^3^) where C is the number of reads that uniquely aligned to gene A, N is the total number of reads that uniquely aligned to all genes, and L is the base number in the coding sequence (CDS) of gene A.

### Phylogenetic tree construction

Protein sequences of interest were aligned with CLUSTAL X [30] and the poorly alignable regions were removed by GBlocks Server at http://molevol.cmima.csic.es/castresana/Gblocks_server.html [31]. PROTTEST was used to select the best fit empirical substitution model of protein evolution based on the Bayesian information criterion [32]. Maximum-likelihood trees were constructed using MEGA software version 6.0.6 [33].

### Data availability

Raw Illumina sequencing data of *L*. *rhinocerotis* TM02 strain was submitted to NCBI Sequence Read Archive (SRA) at http://www.ncbi.nlm.nih.gov/Traces/sra with the accession number SRR1509475 under experiment SRX648275. The Whole Genome Shotgun project of *L*. *rhinocerotis* TM02 strain used in this paper is version AXZM01000000 as previously described [18].

## Results and Discussion

### RNA sequencing assessment

RNA sample extracted from *L*. *rhinocerotis* sclerotium was subjected to high-throughput Illumina sequencing in order to gain an overview of the fungus transcriptome. Over 5 Gb of raw reads were obtained from each cDNA library. The sequencing assessment revealed a good sequencing quality and alignment results. In total, 52,933,332 reads with 90 bp in length were obtained after filtering the raw reads. The clean reads were mapped to the genome and genes sequences of *L*. *rhinocerotis* (Table 1). The mapping results provide an overall assessment of the sequencing. A total of 65.4 and 46.1% clean reads were able to map to the *L*. *rhinocerotis* genome and gene respectively and about 68.0 and 74.0% of the respective mapped reads got the perfect match (without mismatch). Less than 0.5% mapped reads are multi-position match while majority are unique match where the reads were mapped to unique (only one) positions of the genome or gene.

**Table 1:** Alignment statistics of *L*. *rhinocerotis* transcriptome.

	Reads number	Percentage (%)
Total clean reads	52,933,332	100.0
Total base pairs (bp)	4,763,999,880	^__^
**Mapping to genome**		
*Total mapped reads*	34,630,861	65.4
- Perfect match^a^	23,609,560	44.6
- ≤ 2 bp mismatch^a^	9,225,238	17.4
- Unique match^b^	34,385,440	65.0
- Multi-position match^b^	245,421	0.5
*Total unmapped reads*	18,302,471	34.6
**Mapping to gene**		
*Total mapped reads*	24,405,863	46.1
- Perfect match^a^	18,080,611	34.2
- ≤ 3 bp mismatch^a^	6,325,252	12.0
- Unique match^b^	24,229,780	45.8
- Multi-position match^b^	176,083	0.3
*Total unmapped reads*	28,527,469	53.9

^a^ Classified by mismatch.

^b^ Classified by unique-match.

**Fig 1:**
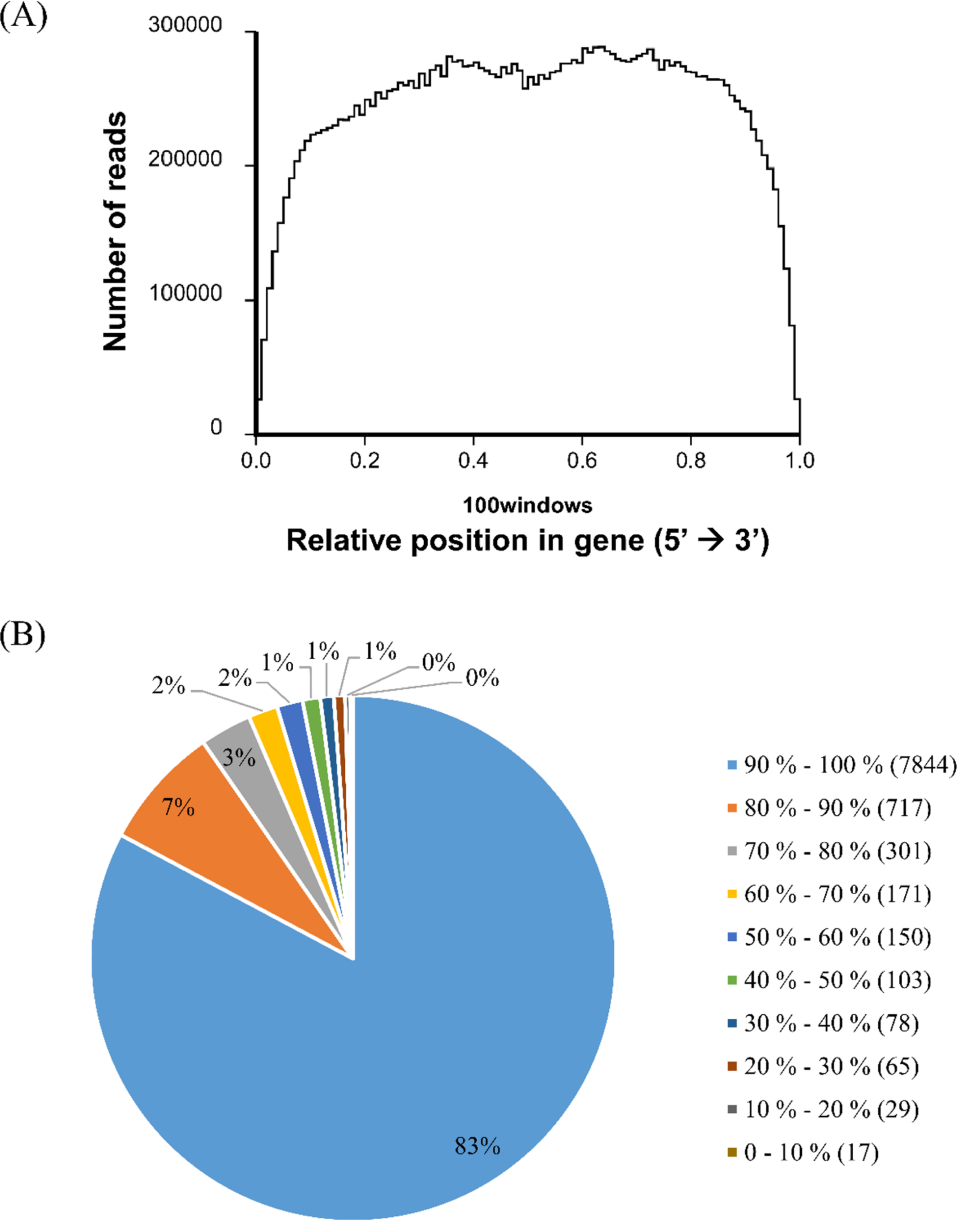
Randomness assessment and gene coverage statistics of *L*. *rhinocerotis*. (A) Distribution statistics of reads mapped to reference gene. The total number of reads aligned to reference genes was counted in the ratio of reads location in reference genes to the length of reference genes. (B) Distribution of genes’ coverage. The percentage of a gene covered by reads equals to ratio of the number of bases in a gene covered by unique mapping reads to number of total bases in that gene.

The randomness of mRNA fragmentation during experimental setting was evaluated with the random distribution of reads in reference genes. A poor randomness of fragmentation leads to generation of reads from specific regions of the original transcripts and affecting subsequent analysis. In this study, the distribution of reads was homogeneous with good randomness of fragmentation (Fig 1A), thus indicating good quality sequence data. Fig 1B shows the distributions of genes’ coverage (the percentage of a gene covered by reads) in *L*. *rhinocerotis*. A total of 88.2% (9,475 of 10,742) of genes were covered by the mapped reads from the transcriptome dataset where 83.0% of the genes showed perfect coverage of 90 to 100%. About 97.0% of genes’ coverage is higher than 50%. A total of 8,641 (91.2%) transcriptome gene models were reassigned upon refinement of gene structures and alternative splicing (AS) analysis.

### AS, Novel transcripts prediction, and SNPs

**Fig 2:**
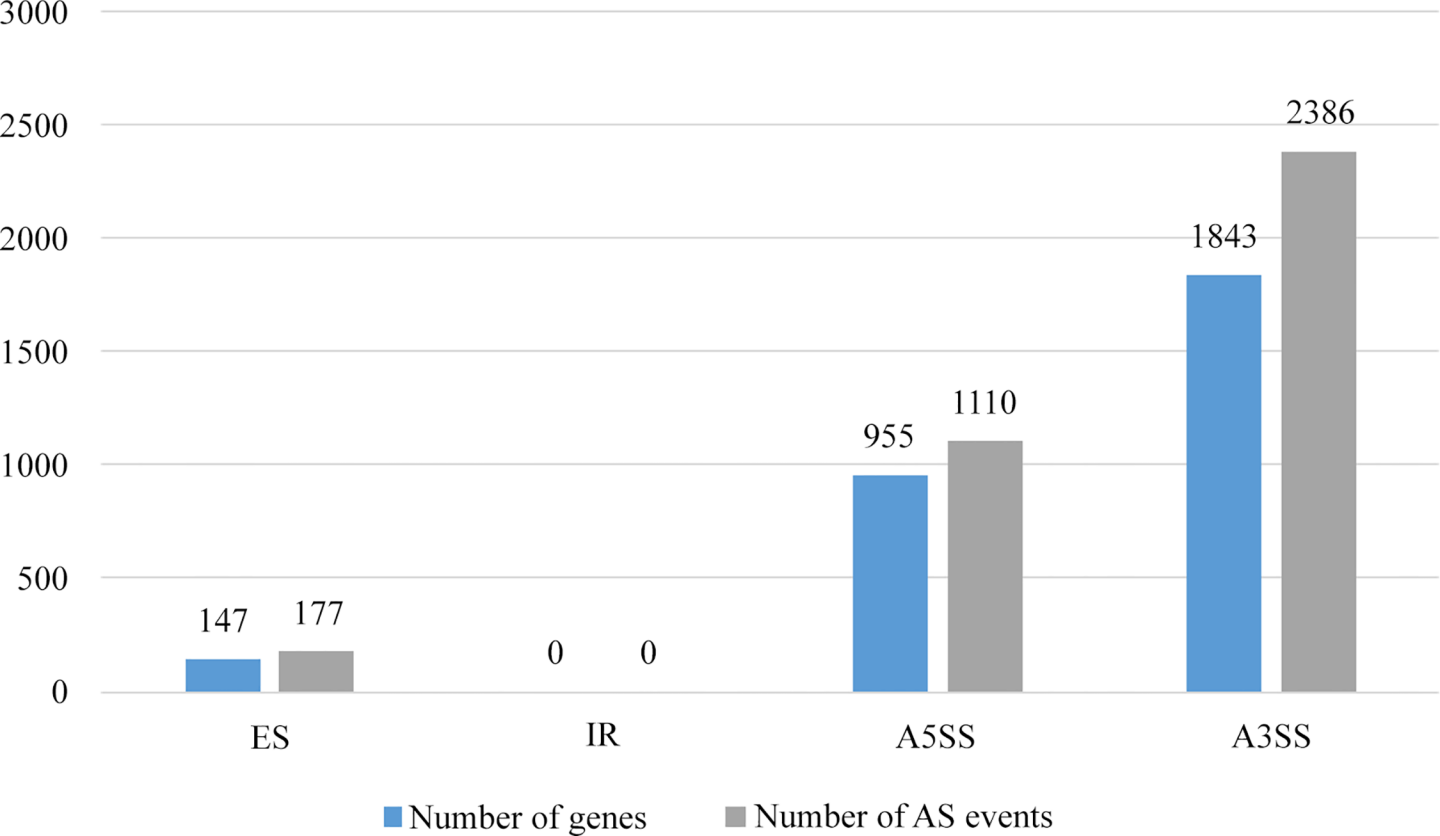
Alternative splicing events and genes in *L*. *rhinocerotis*. Abbreviations: ES, exon skipping; IR, intron retention; A5SS, alternative 5' splice site; A3SS, alternative 3' splice site.

AS is a universal, post-transcriptional event in eukaryotes to increase protein diversity and functional complexity [34]. The seven known modes of AS are exon skipping (ES), intron retention (IR), alternative 5' splice site (A5SS), alternative 3' splice site (A3SS), alternative first exon, alternative last exon, and mutually exclusive exon. The last three modes of AS that produce high false positive results with TopHat were not examined in this study. A total of 3,673 AS events involving 2,497 genes were identified. It is worth noting that some genes produced two or more AS events (Fig 2, S1 Table). A3SS is the major mode of AS in *L*. *rhinocerotis* which makes up 65.0% of the total events. While unlike other fungi such as *Cordyceps militaris* and *Tuber melanosporum*, IR was not detected in *L*. *rhinocerotis* [21, 35]. Several genes presented all three modes of AS, such as those coding for oxidoreductase (GME10159_g), aldehyde dehydrogenase (GME4106_g), peroxiredoxin Q (GME1120_g), and cytochrome P450 (GME4821_g).

Novel transcripts can be found via high throughput sequencing to enrich the present database which may be incomplete. A novel transcript is not a known gene but its expressed depth is at least 2. A total of 117 novel transcripts with 244 exons were predicted in *L*. *rhinocerotis* and about 28.2% of them were longer than 500 bp (S2 Table). The novel transcripts were mostly found in scaffold 1, 10, 100 with expression level (reads number per bp, RNPB) ranged from minimum 2 to highest 164.67.

SNP variation of *L*. *rhinocerotis* was investigated with SOAPsnp based on the Bayes’ theorem (the reverse probability model) to call consensus genotype by carefully considering the data quality, alignment, and recurring experimental errors [24, 36]. A total of 59,649 SNP events which account an average SNP frequency of merely 0.2% (59,649/34,316,739) per nucleotide involving 712 scaffolds were identified (S3 Table). About 56.2% (33,492) of the SNPs fall within the CDS of 6,857 genes and 13,359 SNPs in the coding region of 5,102 genes are non-synonymous. Of them, 63 (0.5%) and 33 (0.3%) are nonsense and “nonstop” mutations, respectively. “Nonstop” mutation occurs within a stop codon and leads to the continued and inappropriate translation of the mRNA into the 3'-untranslated region [37].

### Genome-based functional annotation of *L*. *rhinocerotis* transcriptome

**Fig 3:**
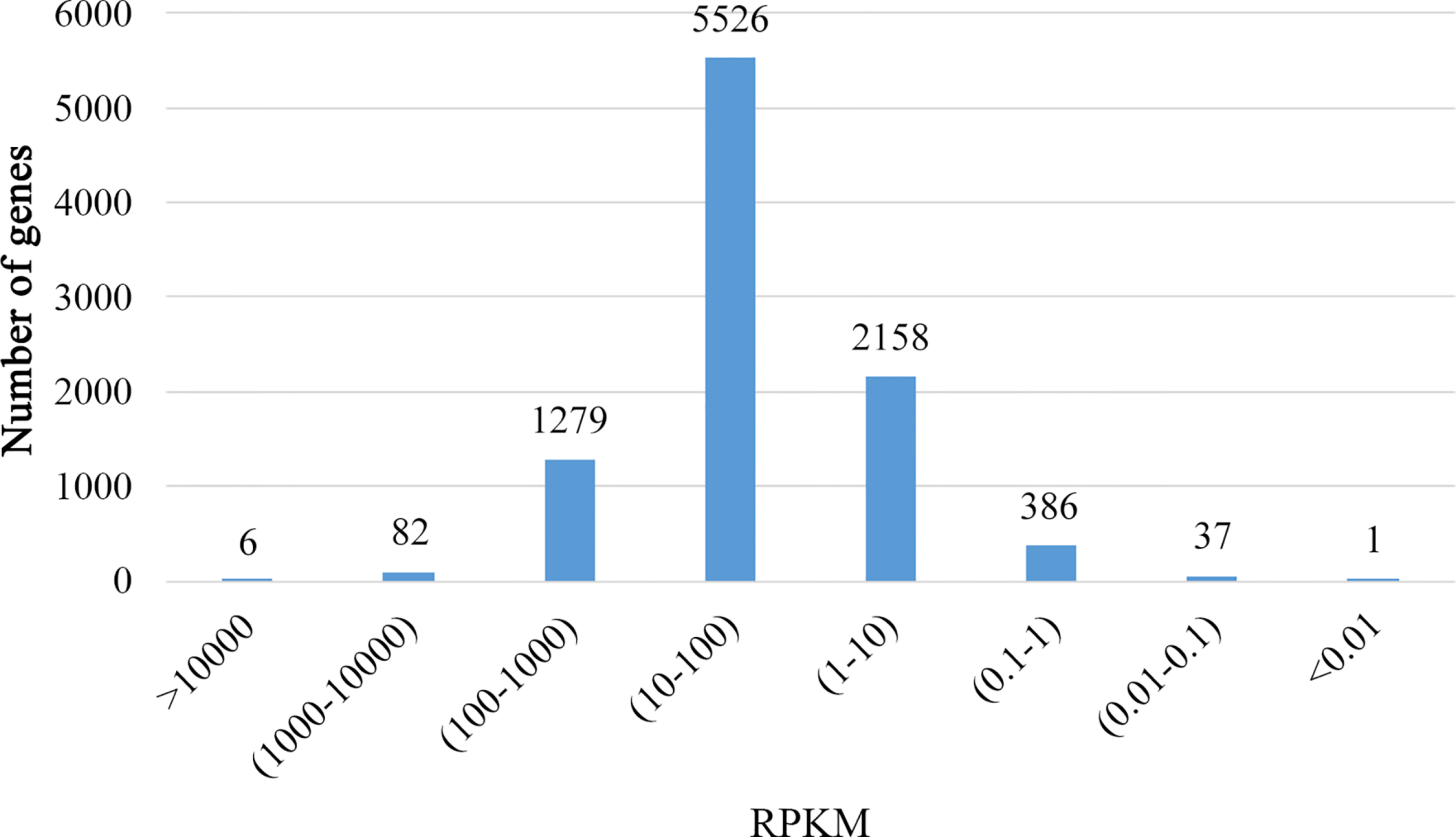
Gene expression distribution of *L*. *rhinocerotis* transcriptome. Genes were grouped into eight log-scaled bins according to their expressions. Abbreviation: RPKM, reads per kilobase per million reads.

Gene expression was calculated using RPKM. The longest transcript for a gene was used to calculate its expression level and coverage when there is more than one transcript. Fig 3 shows the gene expression distribution in *L*. *rhinocerotis* transcriptome. The genes were grouped into eight log-scaled bins according to their expressions and they are considered to be lowly (or highly) expressed if their RPKMs are below 1 (or above 100); 4.5% of the genes are lowly expressed while 14.4% were considered highly expressed with RPKMs above 100.

Genome-based functional annotation revealed that 8,377 (88.4%) of the 9,475 genes covered by the reads from transcriptome dataset were significantly similar to known proteins in the NCBI nr database where more than 75.0% of the 8,377 genes were non-informative with unknown function and/or uncharacterized yet. For instance, “predicted”, “unnamed”, “putative”, “expressed”, and “hypothetical” proteins. The e-value distribution of the top hits in the NCBI nr database showed that 72.5% of the mapped sequences exhibited strong homology (≤ 1e-50), while the e-value distribution of the remaining 27.5% homologous sequences ranged between 1e-5 and 2e-50. S4 and S5 Tables list the top 100 highly expressed annotated genes and top 100 highly expressed genes with informative annotations, respectively. GME3505_g, GME1052_g, and GME275_g that encode for respective cerato-platanin (CP), fungal hydrophobin, and lectin are among the genes with notable expression values (5,842.62–65,892.65 RPKM). Their structural and functional features were further discussed in a later sub-section. Comparison against the SwissProt database yielded a smaller percentage of matches with 5,684 (60.0%) significantly similar BLAST hits and 47.6% of them exhibited strong homology (≤ 1e-50). TrEMBL database returned a comparable percentage of matched genes to NCBI nr, 8,383 (88.5%) hits with 6,071 (72.4%) of them exhibited strong homology (≤ 1e-50). Altogether, 8,390 genes were assigned with descriptions by comparison with NCBI nr, SwissProt, and TrEMBL databases (data not shown).

The transcriptomics data was correlated with *L*. *rhinocerotis* proteomic dataset from our recent work [38] by comparing the mRNA abundance (in RPKM) and protein abundance (in peptide counts per kilobase). A total of 378 identified non-redundant proteins with complete data for the pair of variables (“Distinct peptide” > 0, RPKM > 0) were compared (S1 Fig). In particular, the putative lectins and CP were found to be abundantly expressed in the proteome of *L*. *rhinocerotis* sclerotium, which correlated with the transcriptome analysis. Two serine proteases, which account for 11.1% of the total proteome are encoded by genes GME4347_g (10.5%) and GME8711_g (0.6%) which were expressed at considerable level with RPKM values of 2,693.41 and 1,173.79, respectively [39]. Interestingly, a cytotoxic F5 fraction which was identified to be serine protease encoded by GME4347_g was found to exhibit potent selective cytotoxicity against human breast adenocarcinoma (MCF7) cells with IC_50_ value of 3.00 ± 1.01 μg/ml [38].

In general, the protein abundance dataset (n = 378) had a modest positive correlation of 0.4290 with mRNA expression (*p* < 0.05). The relationship is not in a perfect association where for many of the values of RPKM, it appears that protein abundance (peptides/kbp genes) may be either low or high. An adjusted squared Pearson correlation coefficient of 0.1819 suggested that about 20% of the variation in protein abundance can be explained by mRNA expression levels (S1 Fig). Majority of the expressed proteins have RPKMs value of 100 to 1,000. The relatively poor correlation is not entirely surprising. Although transcriptome has always been perceived as a precursor for proteome, mRNA expression and corresponding protein do not necessarily follow linearity. This could be due to the differences in stability and turnover of mRNAs and proteins as well as various cellular regulatory processes such as transcriptional, post-transcriptional, translational, and protein degradation in controlling steady-state protein abundances [40]. Protein identification is further affected by the type of protein sample preparation methods used. For instance, the approach employed in our recent studies [38, 39] which involved the elution of proteins from polyacrylamide gel electrophoresis is generally limited to proteins that are not too hydrophobic and within the molecular weight ranges of 6 to 202 kDa [41, 42]. Nevertheless, the transcriptomics data in this study will complement the proteomic dataset in understanding the biology and pharmacological properties of this medicinal mushroom.

**Fig 4:**
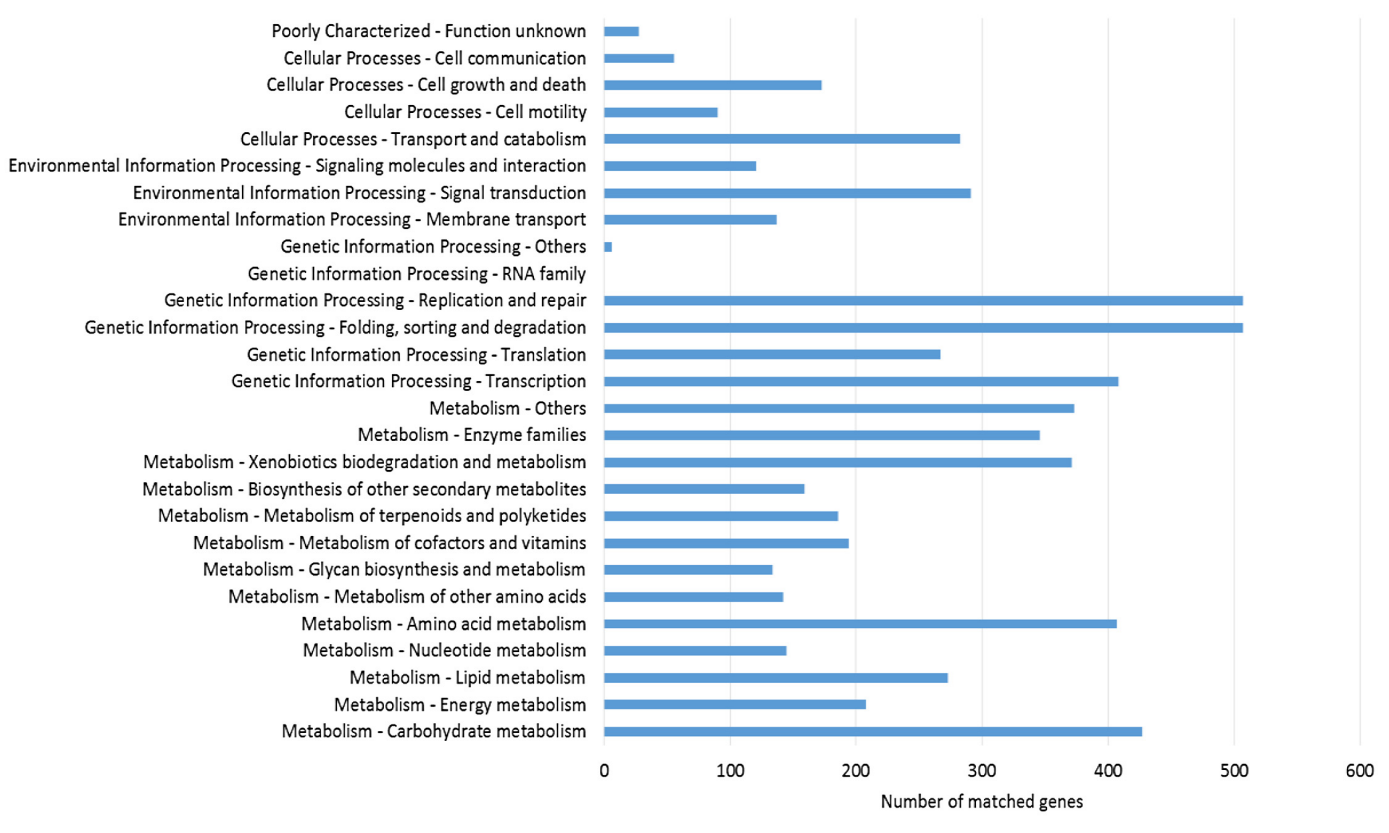
KEGG pathways classification of *L*. *rhinocerotis* transcriptome.

The genes covered by the reads from transcriptome dataset were mapped to KEGG to identify the biological pathways that are active in *L*. *rhinocerotis*. A total of 4,824 (50.9%) genes were assigned to the KEGG orthology (KO) system. About 1.7% (84) of them are lowly expressed with RPKMs below 1. Thus, only the moderately to highly expressed mapped genes (RPKMs above 1) were considered for downstream analysis. Fig 4 shows the representation of the KEGG pathways in first and second layers. The identified KEGG pathways provide a research platform for *L*. *rhinocerotis* metabolic pathways, particularly its secondary metabolism as described previously [18]. One gene could be annotated into more than one KO term. KEGG annotations of *L*. *rhinocerotis* transcriptome showed that carbohydrate and amino acid metabolisms were active in this mushroom.

**Fig 5:**
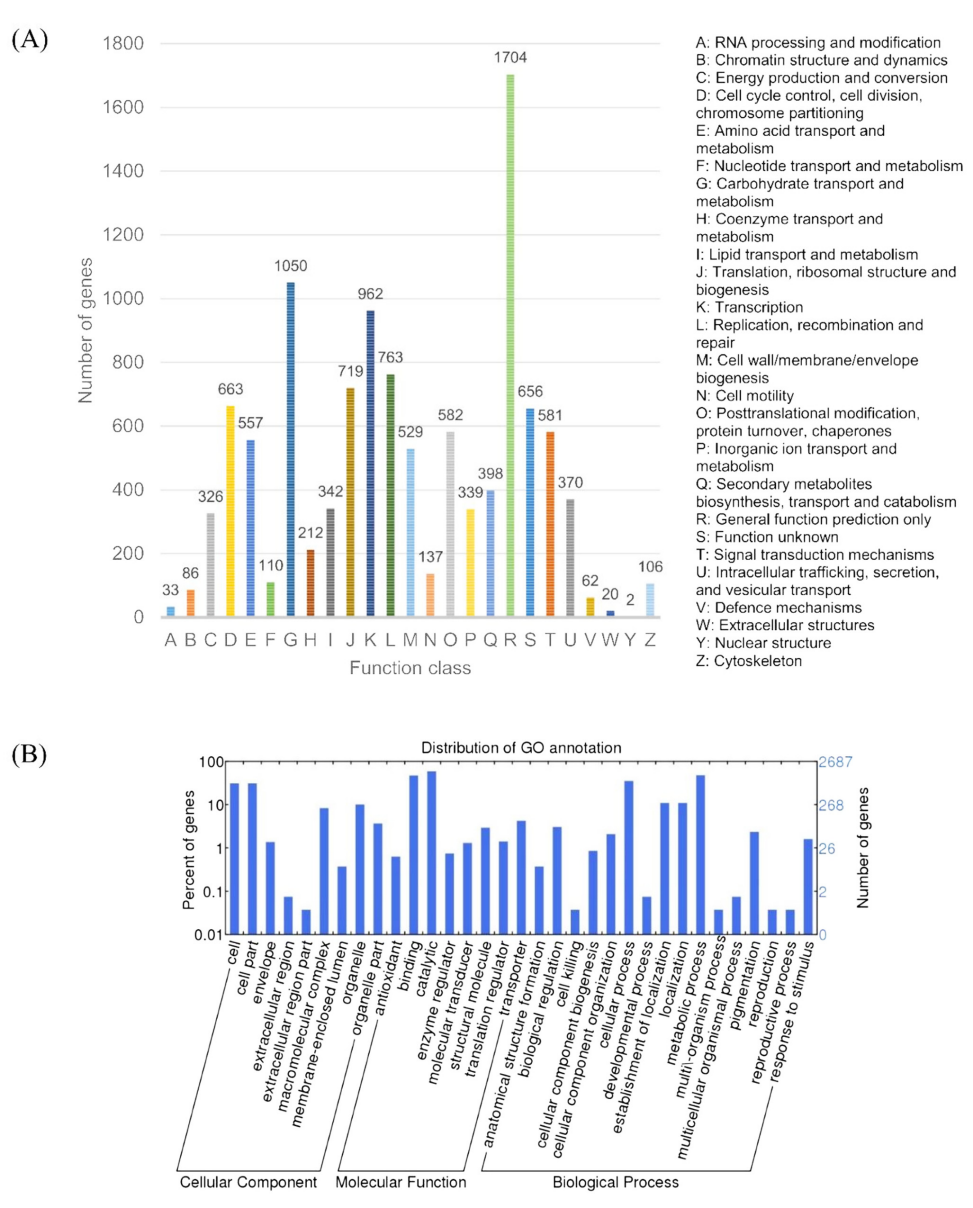
COG and GO annotations of *L*. *rhinocerotis* transcriptome. (A) Histogram presentation of COG classification. (B) GO classification. Histogram was generated using web histogram tool WEGO at http://wego.genomics.org.cn/cgi-bin/wego/index.pl.

The annotated sequences for the genes involved in COG classifications were further identified. In total, 4,788 (50.5%) genes were assigned to COG. Only 90 (1.9%) of them are lowly expressed and was not considered in the analysis (Fig 5A). Some genes with multiple functions were classified into more than one COG category. The top ranked R category for “General function prediction only” with 1,704 genes suggested that these genes were not unambiguously assigned to a certain group. A high percentage of genes were from “Carbohydrate transport and metabolism”, “Transcription”, and “Replication, recombination and repair binding” while only a few were categorized to “RNA processing and modification”, “Extracellular structures”, and “Nuclear structure”. KEGG pathways and COG classifications of *L*. *rhinocerotis* transcriptome revealed a large number of expressed genes involved in the processing of gene information (including transcription, translation, replication, and repair) and carbohydrate metabolism. This is comprehensible as some of the expressed high-abundance proteins such as lectins and serine proteases as previously reported are related to carbohydrate-binding activity and post-translational modification, protein turnover, as well as acting as chaperones, respectively [39].

A total of 2,705 (28.6%) genes were categorized into 33 GO functional groups under three main categories of “Cellular component”, “Biological process”, and “Molecular function” (Fig 5B). The latter occupied the largest proportion (37.0%). A high percentage of genes were from “catalytic”, “binding”, and “metabolic process” while only a few were categorized to “extracellular region part”, “cell killing”, and “reproduction”. These findings are almost similar to *Agrocybe aegerita* and *Lentinula edodes* [43, 44]. Nevertheless, further studies are still needed in order to gain a better insight of the metabolic pathways underlying the sclerotium of *L*. *rhinocerotis*. About 0.7% (18) of the mapped genes are lowly expressed with RPKMs lower than 1 and were not considered in this analysis.

### Secondary metabolite gene clusters from *L*. *rhinocerotis* transcriptome

Some fungal secondary metabolites are known to have interesting bioactivity including antibacterial, antifungal, antiviral, and antitumor [45]. The genes involved in secondary metabolism of *L*. *rhinocerotis* were previously described [18]. In this study, the clustering of genes encoding secondary metabolites was re-examined using the transcriptome resource. Lowly expressed genes with RPKMs lower than 1 were not considered for analysis. A minimum of six gene clusters encoding four terpene synthases, and one each of non-ribosomal peptide synthetase, and polyketide synthase were identified (Table 2). These enzymes are crucial for the biosynthesis of terpenes, peptides such as cyclic peptide antibiotics, and polyketides, respectively. Terpenoids are known to have beneficial pharmaceutical properties. Some renowned fungal terpenoids include pleuromutilin antibiotics, anticancer active illudins, and the ganoderic acids from *Ganoderma lucidum* [46–48]. Present transcriptome study reveals the secondary metabolite pathways that are active in the *L*. *rhinocerotis* sclerotium. This finding will be a useful guide to establish the connection between genes and molecules for compound isolation work where at current, we are attempting to heterologously express these sclerotium-expressed secondary metabolite genes and to further characterise the gene products.

**Table 2:** Secondary metabolite gene clusters.

Cluster	Gene description	Gene start	Gene end	RPKM
*Cluster 1*: *Terpene*				
GME3634_g	terpenoid synthase	30452	34218	23.94
GME3638_g	terpenoid synthase	44397	45444	12.45
GME3639_g	aldo/keto reductase	49551	50988	1.37
*Cluster 2*: *NRP*				
GME6397_g	lysine/ornithine N-monooxygenase	63553	65551	19.84
GME6398_g	nonribosomal peptide synthetase	66567	74891	16.50
*Cluster 3*: *PK*				
GME5065_g	FAD/NAD(P)-binding domain-containing protein	5012	7259	30.99
GME5066_g	polyketide synthase	10525	16200	17.54
*Cluster 4*: *Terpene*				
GME8125_g	terpenoid synthase	24402	25693	2.60
*Cluster 5*: *Terpene*				
GME7269_g	terpenoid synthase	21024	22105	2.05
GME7270_g	terpenoid synthase	23484	24818	1.07
GME7271_g	terpenoid synthase	26316	27822	4.39
*Cluster 6*: *Terpene*				
GME9206_g	NAD-P-binding protein	6545	7927	14.93
GME9207_g	ABC transporter	8535	13055	68.88
GME9210_g	terpenoid synthase	18485	19746	11.20

Abbreviations: RPKM, reads per kilobase per million reads; NRP, non-ribosomal peptide; PK, polyketide.

Cytochrome P450 (CYP) superfamily is a diverse group of enzymes involved in various physiological processes [49]. It was found that *L*. *rhinocerotis* genome had a total of 136 CYP sequences which can be classified into 37 families [18]. However, 22 of them were found to be not transcribed and five were lowly expressed (RPKMs below 1). The remaining 109 CYP sequences were classified into 32 families and ranged from 1.08 to 936.99 RPKM (S6 Table). CYP5144 family had the most number of genes (27 genes). This is followed by CYP5150 (13 genes) and CYP5037 (11 genes) families. These three CYP families are also highly enriched in other basidiomycete fungi including *Phanerochaete chrysosporium*, *Phanerochaete carnosa*, *Agaricus bisporus*, *Postia placenta*, *Ganoderma* sp., and *Serpula lacrymans*. Of all, CYP5144 family forms the largest P450 contingent family [50]. This family is associated with the metabolism of fungal steroids and xenobiotics [51, 52]. *Aspergillus* sp. and *Fusarium verticillioides* which also have the largest numbers of CYP5144 family are known prolific producers of various secondary metabolites [53].

### Insights into the highly-expressed genes in *L*. *rhinocerotis* sclerotium

The genes encoding several small secreted cysteine-rich proteins (CPs and hydrophobins) and lectins were among the highest expressed genes in the sclerotium of *L*. *rhinocerotis*. Several fungal proteins belong to these families have been shown to exhibit desirable bioactivities in previous studies. We performed additional bioinformatics and phylogenetic analyses with comparison to characterised genes/proteins to provide further insights into the functions and taxonomic distribution of these highly-expressed genes in *L*. *rhinocerotis* sclerotium.

### Cerato-platanins (CPs)

**Fig 6:**
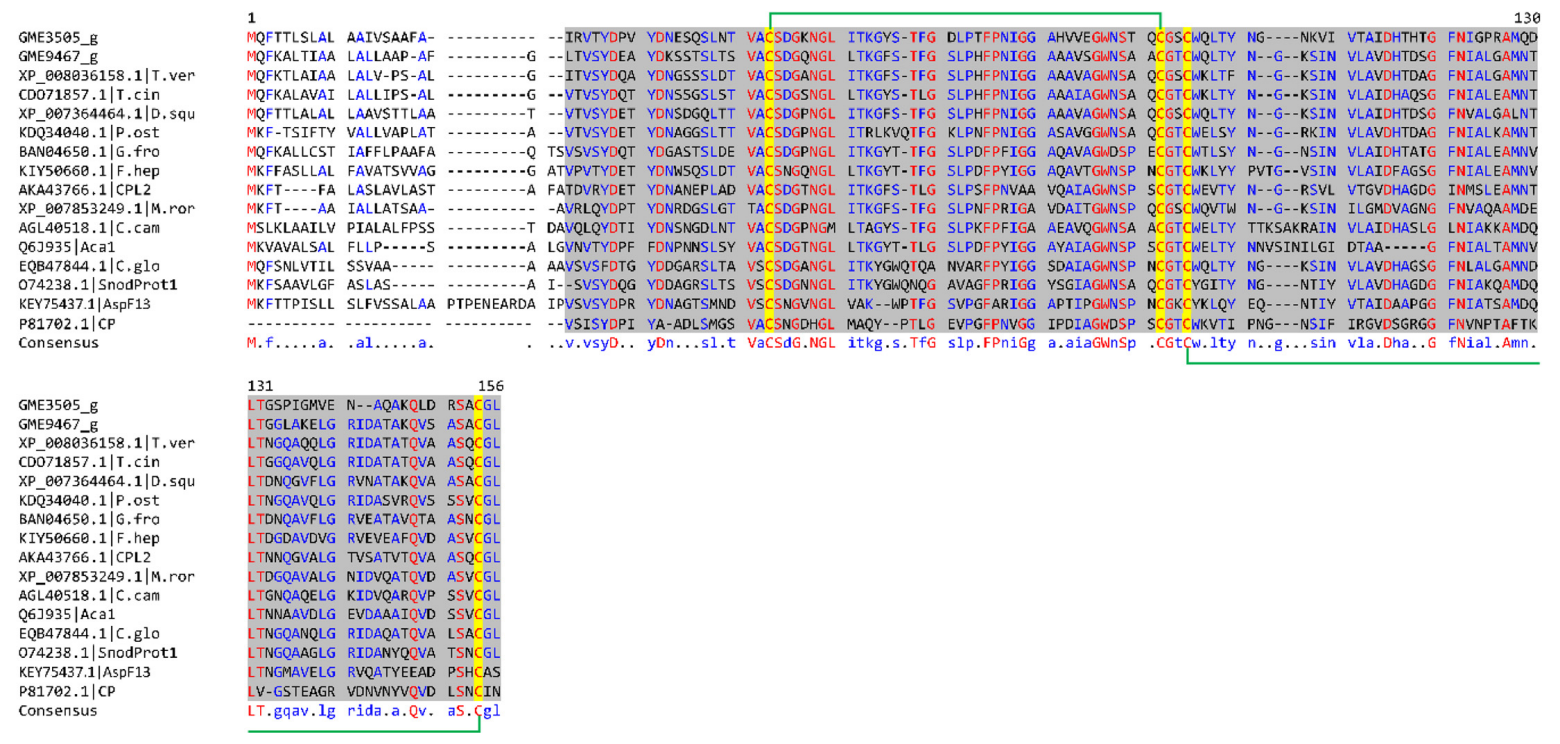
Alignment of putative cerato-platanins from *L*. *rhinocerotis* to cerato-platanin (CP) domain-containing protein sequences. The interval of CP domains are shaded in grey. Residues in red are consensus (100%). Four cysteine residues within the CP domain (highlighted in yellow) are S—S-bonded as indicated by the green brackets. The CP domain-containing proteins were from *Trametes versicolor* (XP_008036158.1), *T*. *cinnabarina* (CDO71857.1), *Dichomitus squalens* (XP_007364464.1), *Pleurotus ostreatus* (KDQ34040.1), *Grifola frondosa* (BAN04650.1), *Fistulina hepatica* (KIY50660.1), *Heterobasidion annosum* (CPL2, AKA43766.1), *Moniliophthora roreri* (XP_007853249.1), *Crinipellis campanella* (AGL40518.1), *Taiwanofungus camphoratus* (Aca1, Q6J935), *Colletotrichum gloeosporioides* (EQB47844.1), *Phaeosphaeria nodorum* (SnodProt1, O74238.1), *Aspergillus fumigatus* (AspF13, KEY75437.1), and *Ceratocystis platani* (CP, P81702.1).

The high expression of several genes in the CP domain containing family correlated well with our proteomic data [38, 39] (Table 3). Among the highly expressed genes, GME3505_g with 65,892.65 RPKM was identified from shotgun proteomics with 2.26% abundance [38]. The CP family conserved domain spans from 20 to 136 residues of the protein. In addition to GME3505_g, a putative protein encoded by GME9467_g with 2,042.84 RPKM and 0.06% protein abundance was also found to carry a CP domain from 21 to 139 residues (Table 3). The CP domain was found to be homologous to an immunomodulatory protein of *Trametes versicolor* FP-101664 SS1 (XP_008036158.1) with 79.0% identity [54]. Multiple sequence alignment of GME3505_g and GME9467_g with their close homologs and several characterised CP family members revealed the four conserved cysteine residues and two conserved tryptophan residues which are hydrophobic (Fig 6). The two disulphide bonds characteristic of CPs were presumably formed between the four conserved cysteine residues of GME3505_g at positions 20, 58, 61, and 115 (Fig 6). For GME9467_g, the disulphide bonds were formed at Cys-21—Cys-59 and Cys-62—Cys-118. Among the aligned CP domain-containing proteins, 14 of them (inclusive of GME3505_g and GME9467_g) contain the CSD amino acid signature sequence while both AspF13 antigen isolated from *Aspergillus fumigatus* and the phytotoxic CP from ascomycete *Ceratocystis fimbriata* f. sp. *platani* contain the CSN signature [55].

**Table 3:** List of selected highly expressed genes in *L*. *rhinocerotis*.

Gene ID	Length (aa)	RPKM	Conserved domain	Accession	Interval (aa)	e-value
GME3505_g	136	65,892.65	Cerato-platanin	pfam07249	20–136	3.82e-45
GME9467_g	139	2,042.84	Cerato-platanin	pfam07249	21–139	2.42e-54
GME1052_g	112	11,012.80	Hydrophobin	pfam01185	31–111	8.81e-23
GME1560_g	261	4,334.98	Hydrophobin	pfam01185	171–259	2.54e-10
GME10149_g	111	3,501.20	Hydrophobin	pfam01185	31–109	1.46e-15
GME2954_g	121	2,928.36	Hydrophobin	pfam01185	62–115	9.02e-12
GME6443_g	139	2,064.56	HYDRO	smart00075	64–118	5.78e-17
GME275_g	147	5,842.62	Lectin/Jacalin-like	cd09612	56–119	5.04e-04
GME273_g	598	3,853.59	Lectin/Jacalin-like	cd09612	272–374	7.90e-06
GME272_g	173	1,198.14	Lectin/Jacalin-like	cd09612	47–139	1.31e-03

Abbreviations: aa, amino acids; RPKM, reads per kilobase per million reads.

**Fig 7:**
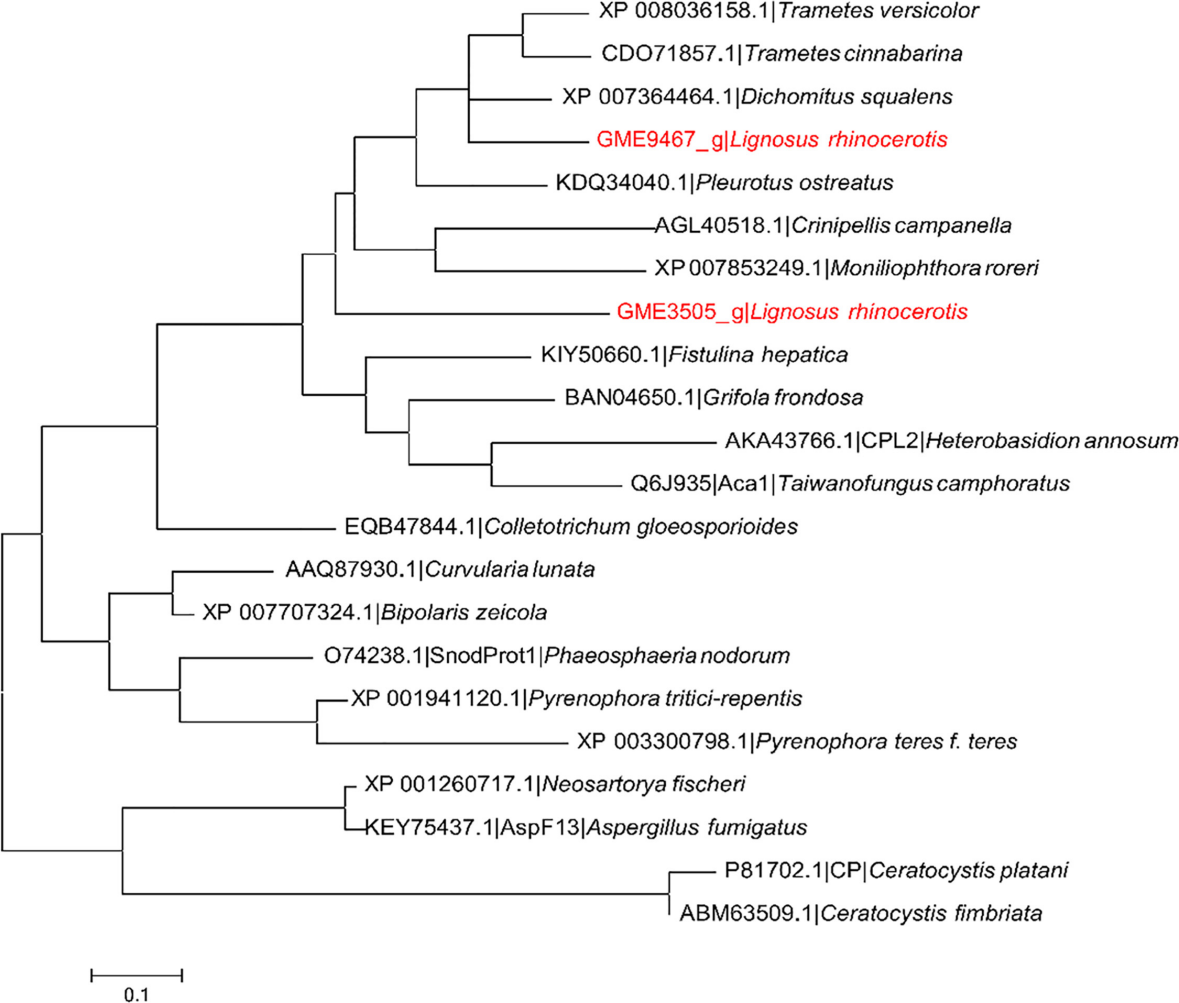
Phylogenetic analysis between putative cerato-platanins from *L*. *rhinocerotis* and related proteins. Maximum-likelihood tree with the highest log likelihood (-2004.2139) is shown. WAG model based on the Bayesian information criterion (BIC) was used as the amino acid substitution model.

CP family proteins have been reported to exhibit various biological activities and their biological roles remain unclear. Some CPs were found to act as allergens while others are elicitors and phytotoxins [56]. Baccelli [57] proposed that the fungal specific CP family proteins exhibit expansin-like activity during fungus-plant interactions and may play multiple biological roles associated to the fungal growth and development, parasitism, cell wall morphogenesis, immune response, and chemotaxis [58]. Phylogenetic analysis of close homologs of CP family members along with previously reported CPs shows that they can be grouped in two major clades corresponding to their origins from basidiomycetes or ascomycetes (Fig 7). The ascomycetes clade was further divided into two groups. As expected, both CP domain-containing proteins from *L*. *rhinocerotis* clustered together with the rest of basidiomycete sequences including CPL2 and the novel immunomodulatory protein Aca1 from *Heterobasidion annosum* and *Taiwanofungus camphoratus* (synonym to *Antrodia camphorata*) respectively but did not cluster with SnodProt1 (from *Phaeosphaeria nodorum*, synonym: *Parastagonospora nodorum*) [59] and CP [60] that have virulence implications as well as the allergenic AspF13 [61]. GME9467_g was found to cluster with the CP domain-containing sequences of *Trametes* sp. and *Dichomitus squalens* that corresponded to their taxonomic hierarchy, which was consistent with our previous phylogeny study [18]. This suggests that GME9467_g homologs are conserved among the Polyporaceae family. On the other hand, GME3505_g which shares 68.3% identity and 75.6% similarity to GME9467_g was found to be relatively distant away from its sub-group.

Among the homologs that clade together with the *L*. *rhinocerotis* proteins, CPL2 was reported to act as an elicitor of defence responses and/or effector of resistance on host and non-host plants [62] while Aca1 possessed immunomodulatory activity by exhibiting TLR2-dependent NF-κB activation and M1 polarization within murine macrophages [63]. Considering this, the putative CP-encoded genes of *L*. *rhinocerotis* may possess similar biological activities, which may contribute to the medicinal properties of this mushroom.

### Fungal hydrophobins

CP was found to be closely related to hydrophobins although it does not contain the unique eight-cysteine pattern characteristic of the latter. Both protein families possess variable degrees of hydrophobicity and accumulates abundantly on the fungal cell surface [60]. The small-to-medium sized hydrophobins (75–120 amino acids) are found in many saprophytic and/or pathogenic fungi [64]. They play prominent roles in fungal morphogenesis, pathogenicity, and host specificity [65]. Putative fungal hydrophobins in *L*. *rhinocerotis* sclerotium are encoded by GME1052_g, GME1560_g, GME10149_g, GME2954_g, and GME6443_g; all with RPKMs above 1,000 (Table 3). The annotated hydrophobins may have similar roles. These genes carry either a hydrophobin (pfam01185) or HYDRO (smart00075) domain. Both domains are members of the hydrophobin superfamily (cl02451). High hydrophobicity of the gene products was found to be incompatible with our previous approaches for protein identification where their abundances were not detected [38, 39]. A different method to isolate hydrophobic proteins should be used in the future to identify these proteins.

**Fig 8:**
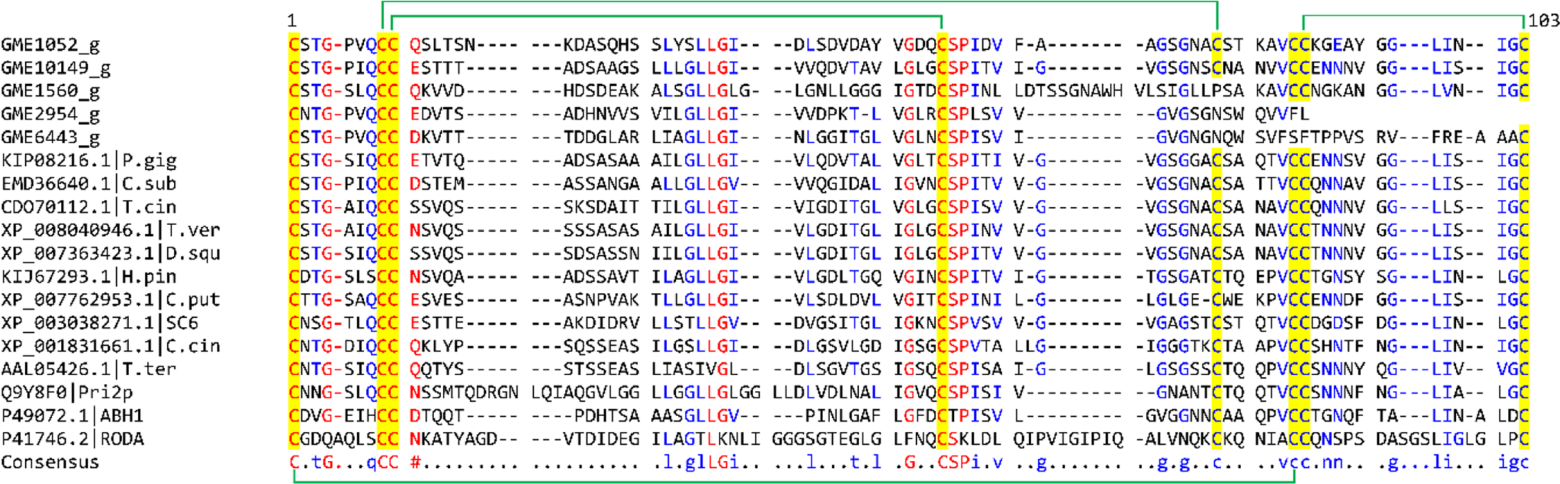
Sequence alignment of hydrophobin domain region within the putative hydrophobin-encoding genes of *L*. *rhinocerotis* and other fungi. Residues in red are highly consensus (90%). Blue coloured fonts indicate low consensus residues (50% less than the first value) and residues in black are neutral. Conserved cysteine residues are highlighted in yellow. Green bracket indicates the position of conserved disulphide bonding between two cysteine residues. The hydrophobin domain-containing proteins were from *Phlebiopsis gigantean* (KIP08216.1), *Ceriporiopsis subvermispora* (EMD36640.1), *Trametes cinnabarina* (CDO70112.1), *Trametes versicolor* (XP_008040946.1), *Dichomitus squalens* (XP_007363423.1), *Hydnomerulius pinastri* (KIJ67293.1), *Coniophora puteana* (XP_007762953.1), *Schizophyllum commune* (SC6, XP_003038271.1), *Coprinopsis cinerea* (XP_001831661.1), *Tricholoma terreum* (AAL05426.1), *Agrocybe aegerita* (Pri2p, Q9Y8F0), *Agaricus bisporus* (ABH1, P49072.1), and *Aspergillus fumigatus* (RODA, P41746.2).

Multiple sequence alignment of the genes to other relatively close amino acid sequences and a few class I hydrophobin family members yielded a mean distance of 1.2137 with overall disparity index of 0.1663 (Fig 8). The gene sequences within the hydrophobin domain region were found to contain four to eight conserved cysteine residues. However, only protein primary structures of GME1052_g and GME10149_g show the C-CC-C-C-CC-C unique pattern of eight cysteine residues forming four conserved disulphide bonds. Nevertheless, sequence conservation between the genes is relatively low. The hydrophobin domain regions of GME1560_g, GME2954_g, and GME6443_g contain a tryptophan residue at position 58 instead of the consensus cysteine. GME2954_g and GME6443_g further show partial domain. Thus, their functionality remains questionable.

**Fig 9:**
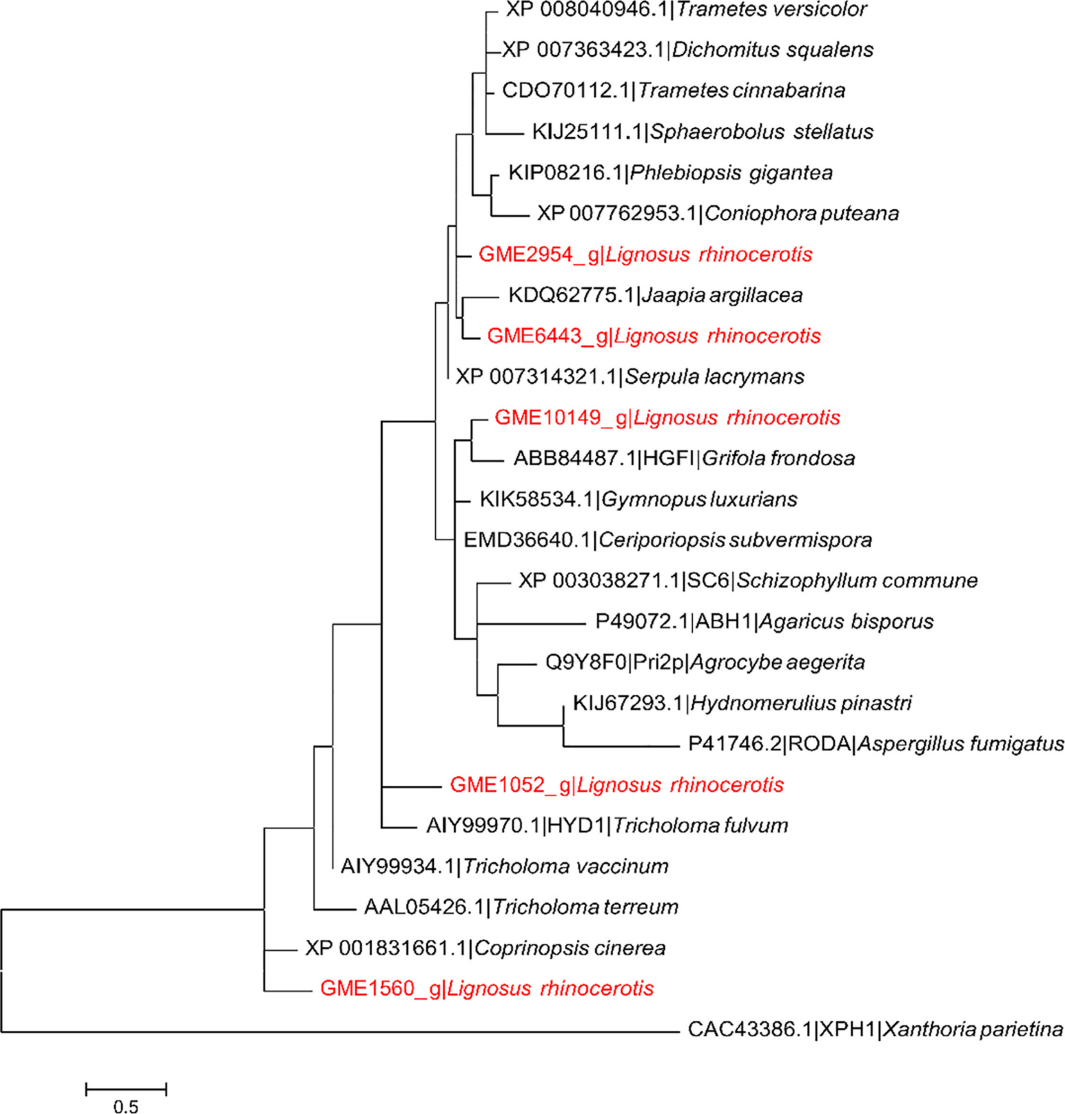
Phylogenetic analysis between putative hydrophobins from *L*. *rhinocerotis* and related proteins. Maximum-likelihood tree with the highest log likelihood (-690.1136) is shown. CpREV model based on the Bayesian information criterion (BIC) was used as the amino acid substitution model.

Phylogenetic analysis of several fungi from the phylum Basidiomycota shows the five hydrophobin domain-containing gene sequences in *L*. *rhinocerotis* are sequentially diverse and their corresponding homologs are distributed across various fungal taxa (Fig 9). Multiple-site indels and variations are likely to account for the differences among the sequences. The two full sequences GME10149_g and GME1052_g show a close genetic linkage to the respective novel 8 kDa HGFI (isolated from *Grifola frondosa*) which forms rodlets in compressed monolayers [66] and HYD1-like protein isolated from *Tricholoma fulvum*. The *hyd1* gene is developmentally regulated in the ectomycorrhizal fungus [67]. Due to the amphipathic nature of these hydrophobins, they have great potential for various biotechnological and medical applications ranging from medical and technical coatings to the production of proteinaceous glue and cosmetics [68].

### Lectins

**Fig 10:**
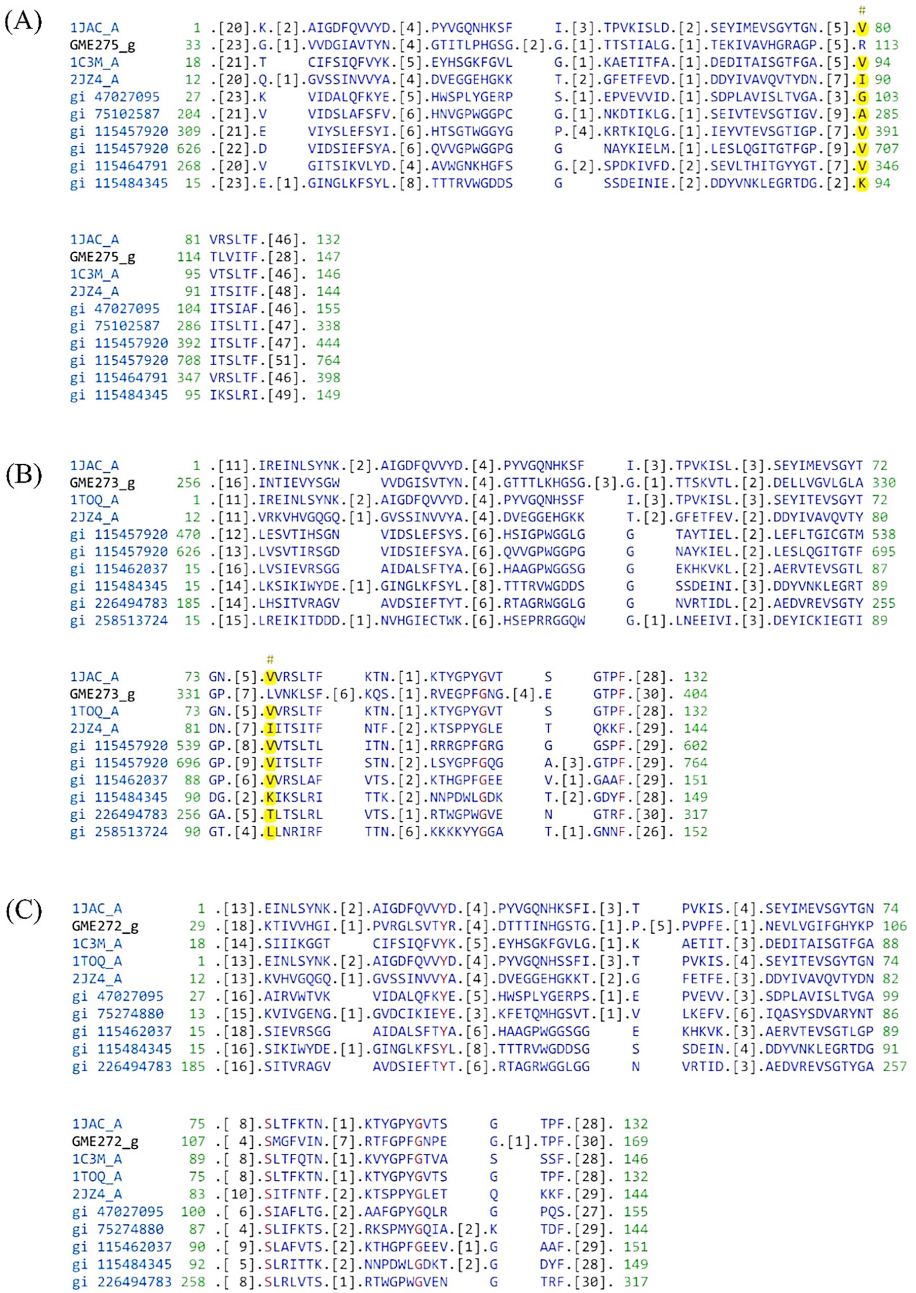
Sequence alignment of lectin-encoding genes’ sugar binding site to top listed sequences from public databases. Residues in red are consensus (100%). Hash marks (#) above the aligned sequences show the location of the conserved feature residues.

In the genome of *L*. *rhinocerotis*, lectins are encoded by highly expressed genes such as GME275_g, GME273_g, and GME272_g (1,198.14–5,842.62 RPKM and some minor ones like GME271_g, GME270_g, GME2199_g, GME2200_g, GME269_g, and GME266_g with RPKMs from 0.76 to 201.79 (Table 3, S5 Table). GME275_g, GME272_g, and GME273_g with protein abundances from 0.29 to 14.96% contain a Jacalin-like plant lectin domain with sugar (chemical) binding site [38]. These Jacalin-like lectins are mostly found in plants and may occur in various oligomerization states. Fig 10 shows the sequence alignment of the genes’ sugar binding site to top listed sequences from public databases. They own a β-prism topology with circularly permuted three-fold repeat of a structural motif and bind mono- or oligosaccharides with high specificity [69]. Unlike plant lectins, the sugar binding site of mushroom lectins show diverse structures, sequences, and carbohydrate recognition properties [70], as supported by the findings from this study. In fact, only a few consensus residues were identified from GME273_g and GME272_g with considerable mutual homology upon sequence alignment to top listed sequences from public databases. On the other hand, GME275_g showed unique sequence (Fig 10).

As these putative lectins encoded by GME275_g, GME272_g, and GME273_g show 36 to 41% identity to Jacalin-type lectin from *G*. *frondosa* (BAE43847.1) [71], they may belong to Group-1 lectins, also known as galectin-like lectins, which are characterized by the presence of a galactose binding lectin domain but with large sequence diversity among the members [70]. Prototype galectin fold consists of a β-sandwich formed by two parallel six stranded antiparallel β-sheets with either one of them forming a concave surface for the binding of β-galactosides [72]. Therefore, it is likely that lectins from *L*. *rhinocerotis* are all-β proteins and may have similar fold to Jacalin and/or galectin. Further investigations like X-ray crystallography, nuclear magnetic resonance spectroscopy, and electron microscopy are required in order to determine their exact structures.

Several fungal lectins have been shown to possess potential pharmacological properties such as mitogenic, immunoenhancing, antiproliferative, antitumor, vasorelaxing, and hypotensive activities [73, 74]. For instance, lectin from *G*. *frondosa* fruiting bodies, a close homolog of *L*. *rhinocerotis* lectins, displayed antiproliferative potential against HeLa cells at 25 μg/ml by cross-linking these cell surface glycoconjugates or through immunomodulatory effects [75, 76]. Whether these putative lectins in *L*. *rhinocerotis* TM02 possess similar pharmacological roles awaits further elucidation.

Fungal lectins may also be involved in various physiological functions related to fungal growth, development, dormancy, and morphogenesis by mobilizing sugars (or carbohydrates) via their specific binding sites and also play a part in molecular recognition during early stage of mycorrhization and fungal morphological changes consequent on parasitic infection [77]. They may as well serve as defence proteins like in plants against predators as they may possess toxic activities such as insecticidal, vermicidal or antiviral [72]. For instance, mushroom galectin *Coprinopsis cinerea* CGL2 was found to play a role in the defence against nematode *Caenorhabditis elegans* via binding to specific glycoconjugate of the host [78]. The various putative lectins encoded by *L*. *rhinocerotis* genome may play similar roles. In particular, they may act as passive-defence proteins to accumulate nitrogen and/or carbohydrate reserves considering the life cycle of this mushroom.

### Polysaccharides biosynthesis

Mushroom polysaccharides are mainly present as glucans within different types of glycosidic linkages. They include the 1,3-β-, 1,6-β-, and 1,3-α-glucans. Mushroom glucans were reported to have various potent pharmacological properties [79]. In particular, the antitumor and immunomodulating properties of water-soluble 1,3-β- and 1,6-β-glucans are well reported [79, 80]. Commercial antitumor polysaccharides like lentinan and schizophyllan isolated from respective *L*. *edodes* and *Schizophyllum commune* are 1,3-β-glucans with β-1,6 branching. Genes encoding enzymes involved in the biosynthesis of uridine diphosphate glucose (UDP-glucose) and the medically important β-glucans were identified in *L*. *rhinocerotis* transcriptome (Table 4). They are considerably expressed with RPKMs from 22.52 to 910.75. Biosynthesis of the precursor of glucans, UDP-glucose, involved hexokinase, phosphoglucomutase, and UDP-glucose-1-phosphate uridylyltransferase [44, 81]. Two 1,3-β-glucan synthases and three genes encoding the β-glucan synthesis-associated protein were also discovered. They are crucial for the biosynthesis of 1,3-β- and 1,6-β-glucans, respectively [81, 82]. Others like calnexin, guanosine triphosphatase (GTPase) activating proteins, and rho GTPase activating protein are responsible for the regulation of 1,3-β- and 1,6-β-glucan biosynthesis. They play important roles in cell wall polysaccharide content regulation [83, 84].

**Table 4:** Genes involved in the biosynthesis of polysaccharides in *L*. *rhinocerotis*.

Gene ID	Gene description	Accession number	e-value	Identity (%)	RPKM
GME4395_g	hexokinase [*Trametes versicolor* FP-101664 SS1]	EIW62140.1	0	83	151.13
GME8190_g	hexokinase [*Trametes versicolor* FP-101664 SS1]	EIW52062.1	0	87	172.42
GME3783_g	phosphoglucomutase [*Dichomitus squalens* LYAD-421 SS1]	EJF56569.1	0	90	105.77
GME4726_g	UTP-glucose-1-phosphate uridylyltransferase [*Dichomitus squalens* LYAD-421 SS1]	EJF65102.1	0	95	796.87
GME8650_g	UTP-glucose-1-phosphate uridylyltransferase [*Dichomitus squalens* LYAD-421 SS1]	EJF65182.1	0	93	35.87
GME792_g	1,3-beta-glucan synthase [*Trametes versicolor* FP-101664 SS1]	EIW60373.1	0	92	68.23
GME5720_g	1,3-beta-glucan synthase [*Dichomitus squalens* LYAD-421 SS1]	EJF59836.1	0	92	107.83
GME7824_g	beta-glucan synthesis-associated protein [*Dichomitus squalens* LYAD-421 SS1]	EJF66803.1	0	73	22.52
GME7856_g	beta-glucan synthesis-associated protein [*Dichomitus squalens* LYAD-421 SS1]	EJF66803.1	0	74	90.19
GME7857_g	beta-glucan synthesis-associated protein [*Punctularia strigosozonata* HHB-11173 SS5]	EIN14050.1	0	70	231.02
GME1428_g	calnexin [*Dichomitus squalens* LYAD-421 SS1]	EJF67129.1	0	80	910.75
GME381_g	GTPase-activating protein gyp7 [*Coprinopsis cinerea* okayama7#130]	XP_002911878.1	0	71	32.76
GME5097_g	GTPase activating protein [*Coprinopsis cinerea* okayama7#130]	XP_001831692.2	0	61	30.08
GME7779_g	GTPase activating protein [*Coprinopsis cinerea* okayama7#130]yahoo	XP_001828717.2	0	50	40.17
GME6438_g	rho GTPase activating protein 22 [*Coprinopsis cinerea* okayama7#130]	XP_001829805.2	0	44	61.83

Abbreviations: RPKM, reads per kilobase per million reads.

## Conclusions

Insights into the transcriptome of *L*. *rhinocerotis* is valuable for inferring the functional elements of its genome and for further understanding of the medicinal properties of this mushroom. This is the first transcriptome re-sequencing analysis of *L*. *rhinocerotis* using high-throughput Illumina HiSeq™ platform. The transcriptome analysis revealed the expression of several secondary metabolite biosynthetic pathways (in particular for terpene biosynthesis) and putative genes involved in glucans biosynthesis in the sclerotium of *L*. *rhinocerotis*. Interestingly, several genes encoding the cysteine-rich CPs, hydrophobins, and sugar-binding lectins were among the most highly expressed genes in *L*. *rhinocerotis* sclerotium. The homologs of some of the CPs and lectins in *L*. *rhinocerotis* have been previously implicated to exhibit various bioactivities relevant to human diseases. Data from this study provides a valuable resource for future research and exploitation of this mushroom. Specifically, this study identified several candidate proteins and secondary metabolite pathways that warrant further investigations in terms of their bioactivities. Recombinant expression of some of these secondary metabolite pathways and cysteine-rich proteins for further characterisation is currently underway.

## Supporting Information

S1 Fig Correlation between protein abundance and mRNA expression levels. (TIF)

S1 Table Alternative splicing events and genes in *L*. *rhinocerotis*. ES, exon skipping; A5SS, alternative 5' splice site; A3SS, alternative 3' splice site.
(XLSX)

S2 Table List of novel transcripts identified from *L*. *rhinocerotis* sclerotium by RNA-Seq. (XLSX)

S3 Table List of SNPs identified from *L*. *rhinocerotis* sclerotium by RNA-Seq. (XLSX)

S4 Table List of top 100 highly expressed annotated genes in *L*. *rhinocerotis* transcriptome. (XLSX)

S5 Table List of top 100 highly expressed genes with informative annotations in *L*. *rhinocerotis* transcriptome. (XLSX)

S6 Table List of cytochrome P450 genes identified from *L*. *rhinocerotis* sclerotium by RNA-Seq. (XLSX)

## References

[1] Cui BK, Tang LP, Dai YC. Morphological and molecular evidences for a new species of Lignosus (Polyporales, Basidiomycota) from tropical China. Mycological Progress. 2011;10(3):267–71. 10.1007/s11557-010-0697-y

[2] Núñez M, Ryvarden L. East Asian polypores 2. Polyporaceae s. lato. Synopsis Fungorum 2001;14:170–522.

[3] Huang NL. Identification of the scientific name of hurulingzhi. Acta Edulis Fungi. 1999;06(01):32–4.

[4] Chang YS, Lee SS. Utilisation of macrofungi species in Malaysia. Fungal Diversity. 2004;15:15–22.

[5] Burkill IH, Birtwistle W, Foxworthy FW, Scrivenor JB, Watson JG. A Dictionary of the Economic Products of the Malay Peninsula, Volume 1 Kuala Lumpur, Malaysia: Governments of Malaysia and Singapore by the Ministry of Agriculture and Co-operatives; 1966.

[6] Burkill IH, Haniff M. Malay village medicine. Gardens' Bulletin, Straits Settlements. 1930;6:165–321.

[7] Chan GG. Tiger's milk fungus. The Malayan Forester. 1953;2(2):103.

[8] Lai CKM, Wong KH, Cheung PCK. Antiproliferative effects of sclerotial polysaccharides from *Polyporus rhinocerus* Cooke (Aphyllophoromycetideae) on different kinds of leukemic cells. International Journal of Medicinal Mushrooms. 2008;10(3):255–64. 10.1615/IntJMedMushr.v10.i3.60

[9] Lau BF, Abdullah N, Aminudin N, Lee HB. Chemical composition and cellular toxicity of ethnobotanical-based hot and cold aqueous preparations of the tiger′s milk mushroom (Lignosus rhinocerotis). Journal of Ethnopharmacology. 2013;150(1):252–62. 10.1016/j.jep.2013.08.034 23993912

[10] Lee ML, Tan NH, Fung SY, Tan CS, Ng ST. The antiproliferative activity of sclerotia of Lignosus rhinocerus (Tiger Milk Mushroom). Evidence-Based Complementary and Alternative Medicine. 2012;2012:697603 10.1155/2012/697603 22454675 PMC3292113

[11] Suziana Zaila CF, Farida Zuraina MY, Norfazlina MN, Lek Mun L, Nurshahirah N, Florinsiah L, et al Antiproliferative effect of Lignosus rhinocerotis, the tiger milk mushroom on HCT 116 human colorectal cancer cells. The Open Conference Proceedings Journal. 2013;4(Suppl-2, M16):65–70.

[12] Eik LF, Naidu M, David P, Wong KH, Tan YS, Sabaratnam V. *Lignosus rhinocerus* (Cooke) Ryvarden: A medicinal mushroom that stimulates neurite outgrowth in PC-12 cells. Evid Based Complement Alternat Med. 2012;2012:320308 Epub 2011/12/29. 10.1155/2012/320308 22203867 PMC3235797

[13] Abdul Razak MS. Antinoceceptive of *Lignosus rhinocerus* spp Research Report, Universiti Industri Selangor 2009 Available: http://www.scribd.com/doc/73538759/Antinoceceptive-of-Lignosus-Rhinoserus-Spp

[14] Phan CW, David P, Naidu M, Wong KH, Sabaratnam V. Neurite outgrowth stimulatory effects of culinary-medicinal mushrooms and their toxicity assessment using differentiating Neuro-2a and embryonic fibroblast BALB/3T3. BMC Complementary and Alternative Medicine. 2013;13(1):261 10.1186/1472-6882-13-261 24119256 PMC3852280

[15] Shopana M, Sudhahar D, Anandarajagopal K. Screening of Lignosus rhinocerus extracts as antimicrobial agents against selected human pathogens. Journal of Pharmaceutical and Biomedical Sciences. 2012;18(11):1–4.

[16] Wong KH, Lai CKM, Cheung PCK. Stimulation of human innate immune cells by medicinal mushroom sclerotial polysaccharides. International Journal of Medicinal Mushrooms. 2009;11(3):215–23. 10.1615/IntJMedMushr.v11.i3.10

[17] Wong KH, Lai CKM, Cheung PCK. Immunomodulatory activities of mushroom sclerotial polysaccharides. Food Hydrocolloids. 2011;25(2):150–8. 10.1016/j.foodhyd.2010.04.008.

[18] Yap HY, Chooi YH, Firdaus-Raih M, Fung SY, Ng ST, Tan CS, et al The genome of the tiger milk mushroom, Lignosus rhinocerotis, provides insights into the genetic basis of its medicinal properties. BMC Genomics. 2014;15:635 Epub 2014/07/31. 10.1186/1471-2164-15-635 25073817 PMC4129116

[19] Wong MM, Cannon CH, Wickneswari R. Identification of lignin genes and regulatory sequences involved in secondary cell wall formation in Acacia auriculiformis and Acacia mangium via de novo transcriptome sequencing. BMC Genomics. 2011;12:342 Epub 2011/07/07. 10.1186/1471-2164-12-342 21729267 PMC3161972

[20] Li H, Dong Y, Yang J, Liu X, Wang Y, Yao N, et al *De novo* transcriptome of safflower and the identification of putative genes for oleosin and the biosynthesis of flavonoids. PLoS ONE. 2012;7(2):e30987 10.1371/journal.pone.0030987 22363528 PMC3283594

[21] Tisserant E, Da Silva C, Kohler A, Morin E, Wincker P, Martin F. Deep RNA sequencing improved the structural annotation of the Tuber melanosporum transcriptome. New Phytol. 2011;189(3):883–91. 10.1111/j.1469-8137.2010.03597.x . 21223284

[22] Twine NA, Janitz K, Wilkins MR, Janitz M. Whole transcriptome sequencing reveals gene expression and splicing differences in brain regions affected by Alzheimer's disease. PLoS One. 2011;6(1):e16266 10.1371/journal.pone.0016266 21283692 PMC3025006

[23] Tan CS, Ng ST, Vikineswary S, Lo FP, Tee CS. Genetic markers for identification of a malaysian medicinal mushroom, *Lignosus rhinocerus* (Cendawan Susu Rimau). Acta Horticulturae (ISHS). 2010;859:161–7. doi: http://www.actahort.org/books/859/859_19.htm

[24] Li R, Yu C, Li Y, Lam TW, Yiu SM, Kristiansen K, et al SOAP2: An improved ultrafast tool for short read alignment. Bioinformatics. 2009;25(15):1966–7. 10.1093/bioinformatics/btp336 . 19497933

[25] Trapnell C, Pachter L, Salzberg SL. TopHat: Discovering splice junctions with RNA-Seq. Bioinformatics. 2009;25(9):1105–11. Epub 2009/03/18. 10.1093/bioinformatics/btp120 19289445 PMC2672628

[26] Ashburner M, Ball CA, Blake JA, Botstein D, Butler H, Cherry JM, et al Gene ontology: Tool for the unification of biology. The Gene Ontology Consortium. Nat Genet. 2000;25(1):25–9. 10.1038/75556 10802651 PMC3037419

[27] Koonin EV, Fedorova ND, Jackson JD, Jacobs AR, Krylov DM, Makarova KS, et al A comprehensive evolutionary classification of proteins encoded in complete eukaryotic genomes. Genome Biol. 2004;5(2):R7 10.1186/gb-2004-5-2-r7 14759257 PMC395751

[28] Kanehisa M, Goto S, Kawashima S, Okuno Y, Hattori M. The KEGG resource for deciphering the genome. Nucleic Acids Res. 2004;32(Database issue):D277–80. 10.1093/nar/gkh063 14681412 PMC308797

[29] Mortazavi A, Williams BA, McCue K, Schaeffer L, Wold B. Mapping and quantifying mammalian transcriptomes by RNA-Seq. Nat Methods. 2008;5(7):621–8. 10.1038/nmeth.1226 . 18516045 PMC13303166

[30] Thompson JD, Gibson TJ, Plewniak F, Jeanmougin F, Higgins DG. The CLUSTAL_X windows interface: Flexible strategies for multiple sequence alignment aided by quality analysis tools. Nucleic Acids Res. 1997;25(24):4876–82. Epub 1998/02/28. 9396791 10.1093/nar/25.24.4876 PMC147148

[31] Castresana J. Selection of conserved blocks from multiple alignments for their use in phylogenetic analysis. Mol Biol Evol. 2000;17(4):540–52. . 10742046 10.1093/oxfordjournals.molbev.a026334

[32] Abascal F, Zardoya R, Posada D. ProtTest: Selection of best-fit models of protein evolution. Bioinformatics. 2005;21(9):2104–5. 10.1093/bioinformatics/bti263 . 15647292

[33] Tamura K, Stecher G, Peterson D, Filipski A, Kumar S. MEGA6: Molecular evolutionary genetics analysis version 6.0. Mol Biol Evol. 2013;30(12):2725–9. Epub 2013/10/18. 10.1093/molbev/mst197 24132122 PMC3840312

[34] Black DL. Mechanisms of alternative pre-messenger RNA splicing. Annu Rev Biochem. 2003;72:291–336. Epub 2003/03/11. 10.1146/annurev.biochem.72.121801.161720 . 12626338

[35] Yin Y, Yu G, Chen Y, Jiang S, Wang M, Jin Y, et al Genome-wide transcriptome and proteome analysis on different developmental stages of Cordyceps militaris. PLoS ONE. 2012;7(12):e51853 10.1371/journal.pone.0051853 23251642 PMC3522581

[36] Bayes M, Price M. An essay towards solving a problem in the doctrine of chances. By the late Rev. Mr. Bayes, F. R. S. Communicated by Mr. Price, in a letter to John Canton, A. M. F. R. S. Philosophical Transactions. 1763;53:370–418. 10.1098/rstl.1763.0053

[37] Hamby SE, Thomas NS, Cooper DN, Chuzhanova N. A meta-analysis of single base-pair substitutions in translational termination codons ('nonstop' mutations) that cause human inherited disease. Human Genomics. 2011;5(4):241–64. Epub 2011/06/30. 21712188 10.1186/1479-7364-5-4-241 PMC3525242

[38] Yap HY, Fung SY, Ng ST, Tan CS, Tan NH. Shotgun proteomic analysis of tiger milk mushroom (Lignosus rhinocerotis) and the isolation of a cytotoxic fungal serine protease from its sclerotium. Journal of Ethnopharmacology. 2015 Epub 2015/09/01. 10.1016/j.jep.2015.08.042 . 26320692

[39] Yap HY, Fung SY, Ng ST, Tan CS, Tan NH. Genome-based proteomic analysis of Lignosus rhinocerotis (Cooke) Ryvarden sclerotium. International Journal of Medical Sciences. 2015;12(1):23–31. 10.7150/ijms.10019 . 25552915 PMC4278872

[40] Vogel C, Marcotte EM. Insights into the regulation of protein abundance from proteomic and transcriptomic analyses. Nat Rev Genet. 2012;13(4):227–32. 10.1038/nrg3185 22411467 PMC3654667

[41] Greenbaum D, Colangelo C, Williams K, Gerstein M. Comparing protein abundance and mRNA expression levels on a genomic scale. Genome Biol. 2003;4(9):117 Epub 2003/09/04. 10.1186/gb-2003-4-9-117 12952525 PMC193646

[42] Gygi SP, Rochon Y, Franza BR, Aebersold R. Correlation between protein and mRNA abundance in yeast. Mol Cell Biol. 1999;19(3):1720–30. Epub 1999/02/18. 10022859 10.1128/mcb.19.3.1720 PMC83965

[43] Zhong M, Liu B, Wang X, Liu L, Lun Y, Li X, et al De novo characterization of Lentinula edodes C(91–3) transcriptome by deep Solexa sequencing. Biochem Biophys Res Commun. 2013;431(1):111–5. Epub 2012/12/26. 10.1016/j.bbrc.2012.12.065 . 23266612

[44] Wang M, Gu B, Huang J, Jiang S, Chen Y, Yin Y, et al Transcriptome and proteome exploration to provide a resource for the study of Agrocybe aegerita. PLoS ONE. 2013;8(2):e56686 10.1371/journal.pone.0056686 23418592 PMC3572045

[45] Keller NP, Turner G, Bennett JW. Fungal secondary metabolism—From biochemistry to genomics. Nat Rev Microbiol. 2005;3(12):937–47. Epub 2005/12/03. 10.1038/nrmicro1286 . 16322742

[46] Liu D, Gong J, Dai W, Kang X, Huang Z, Zhang HM, et al The genome of Ganoderma lucidum provides insights into triterpenes biosynthesis and wood degradation [corrected]. PLoS One. 2012;7(5):e36146 10.1371/journal.pone.0036146 22567134 PMC3342255

[47] Schobert R, Knauer S, Seibt S, Biersack B. Anticancer active illudins: Recent developments of a potent alkylating compound class. Curr Med Chem. 2011;18(6):790–807. Epub 2010/12/25. . 21182482 10.2174/092986711794927766

[48] Novak R, Shlaes DM. The pleuromutilin antibiotics: A new class for human use. Current Opinion in Investigational Drugs. 2010;11(2):182–91. Epub 2010/01/30. . 20112168

[49] Cresnar B, Petric S. Cytochrome P450 enzymes in the fungal kingdom. Biochim Biophys Acta. 2011;1814(1):29–35. Epub 2010/07/14. 10.1016/j.bbapap.2010.06.020 . 20619366

[50] Syed K, Shale K, Pagadala NS, Tuszynski J. Systematic identification and evolutionary analysis of catalytically versatile cytochrome p450 monooxygenase families enriched in model basidiomycete fungi. PLoS ONE. 2014;9(1):e86683 10.1371/journal.pone.0086683 24466198 PMC3899305

[51] Chen S, Xu J, Liu C, Zhu Y, Nelson DR, Zhou S, et al Genome sequence of the model medicinal mushroom Ganoderma lucidum. Nature communications. 2012;3:913 10.1038/ncomms1923 22735441 PMC3621433

[52] Ide M, Ichinose H, Wariishi H. Molecular identification and functional characterization of cytochrome P450 monooxygenases from the brown-rot basidiomycete Postia placenta. Arch Microbiol. 2012;194(4):243–53. Epub 2011/09/23. 10.1007/s00203-011-0753-2 . 21938516

[53] Moktali V, Park J, Fedorova-Abrams ND, Park B, Choi J, Lee YH, et al Systematic and searchable classification of cytochrome P450 proteins encoded by fungal and oomycete genomes. BMC Genomics. 2012;13:525 Epub 2012/10/05. 10.1186/1471-2164-13-525 23033934 PMC3505482

[54] Floudas D, Binder M, Riley R, Barry K, Blanchette RA, Henrissat B, et al The Paleozoic origin of enzymatic lignin decomposition reconstructed from 31 fungal genomes. Science. 2012;336(6089):1715–9. Epub 2012/06/30. 10.1126/science.1221748 . 22745431

[55] Chen H, Kovalchuk A, Kerio S, Asiegbu FO. Distribution and bioinformatic analysis of the cerato-platanin protein family in Dikarya. Mycologia. 2013;105(6):1479–88. Epub 2013/08/10. 10.3852/13-115 . 23928425

[56] de OBMR, de Oliveira JF, Adamoski D, Teixeira PJ, do Prado PF, Tiezzi HO, et al Functional diversification of cerato-platanins in Moniliophthora perniciosa as seen by differential expression and protein function specialization. Mol Plant Microbe Interact. 2013;26(11):1281–93. Epub 2013/08/02. 10.1094/mpmi-05-13-0148-r . 23902259

[57] Baccelli I. Cerato-platanin family proteins: One function for multiple biological roles? Frontiers in Plant Science. 2014;5:769 10.3389/fpls.2014.00769 . 25610450 PMC4284994

[58] Yu H, Li L. Phylogeny and molecular dating of the cerato-platanin-encoding genes. Genet Mol Biol. 2014;37(2):423–7. Epub 2014/07/30. 25071408 10.1590/s1415-47572014005000003 PMC4094615

[59] Jeong JS, Mitchell TK, Dean RA. The Magnaporthe grisea SnodProt1 homolog, MSP1, is required for virulence. FEMS Microbiology Letters. 2007;273(2):157–65. 17590228 10.1111/j.1574-6968.2007.00796.x

[60] Pazzagli L, Cappugi G, Manao G, Camici G, Santini A, Scala A. Purification, characterization, and amino acid sequence of cerato-platanin, a new phytotoxic protein from Ceratocystis fimbriata f. sp. platani. J Biol Chem. 1999;274(35):24959–64. Epub 1999/08/24. . 10455173 10.1074/jbc.274.35.24959

[61] Rementeria A, López-Molina N, Ludwig A, Vivanco AB, Bikandi J, Pontón J, et al Genes y moléculas implicados en la virulencia de Aspergillus fumigatus. Revista Iberoamericana de Micología. 2005;22(1):1–23. 10.1016/S1130-1406(05)70001-2 15813678

[62] Chen H, González JQ, Kovalchuk A, Ubhayasekera W, Asiegbu FO, editors. A cerato-platanin-like protein HaCPL2 from *Heterobasidion annosum sensu stricto* induces cell death in *Nicotiana tabacum* and *Pinus sylvestris*. Seminar on Forest Pathology; 2015 April 16th, 2015; Natural Resources Institute Finland, Tikkurila Vantaa. 10.1016/j.fgb.2015.09.007 26385823

[63] Sheu F, Chien PJ, Hsieh KY, Chin KL, Huang WT, Tsao CY, et al Purification, cloning, and functional characterization of a novel immunomodulatory protein from Antrodia camphorata (bitter mushroom) that exhibits TLR2-dependent NF-kappaB activation and M1 polarization within murine macrophages. J Agric Food Chem. 2009;57(10):4130–41. Epub 2009/04/18. 10.1021/jf900469a . 19371137

[64] Wosten HA, de Vocht ML. Hydrophobins, the fungal coat unravelled. Biochim Biophys Acta. 2000;1469(2):79–86. Epub 2000/09/22. . 10998570 10.1016/s0304-4157(00)00002-2

[65] Ebbole DJ. Hydrophobins and fungal infection of plants and animals. Trends in Microbiology. 1997;5(10):405–8. 10.1016/S0966-842X(97)01130-X 9351177

[66] Yu L, Zhang B, Szilvay GR, Sun R, Janis J, Wang Z, et al Protein HGFI from the edible mushroom Grifola frondosa is a novel 8 kDa class I hydrophobin that forms rodlets in compressed monolayers. Microbiology. 2008;154(Pt 6):1677–85. Epub 2008/06/06. 10.1099/mic.0.2007/015263-0 . 18524922

[67] Mankel A, Krause K, Kothe E. Identification of a hydrophobin gene that is developmentally regulated in the ectomycorrhizal fungus Tricholoma terreum. Applied and Environmental Microbiology. 2002;68(3):1408–13. 10.1128/AEM.68.3.1408-1413.2002 . 11872494 PMC123729

[68] Hektor HJ, Scholtmeijer K. Hydrophobins: Proteins with potential. Curr Opin Biotechnol. 2005;16(4):434–9. Epub 2005/06/14. 10.1016/j.copbio.2005.05.004 . 15950452

[69] Sankaranarayanan R, Sekar K, Banerjee R, Sharma V, Surolia A, Vijayan M. A novel mode of carbohydrate recognition in jacalin, a Moraceae plant lectin with a beta-prism fold. Nat Struct Biol. 1996;3(7):596–603. Epub 1996/07/01. . 8673603 10.1038/nsb0796-596

[70] Khan F, Khan MI. The mushroom lectins show three types of conserved domain in a bioinformatics analysis. American Journal of Biochemistry and Molecular Biology. 2011;1:375–88.

[71] Nagata Y, Yamashita M, Honda H, Akabane J, Uehara K, Saito A, et al Characterization, occurrence, and molecular cloning of a lectin from Grifola frondosa: jacalin-related lectin of fungal origin. Biosci Biotechnol Biochem. 2005;69(12):2374–80. Epub 2005/12/27. 10.1271/bbb.69.2374 . 16377896

[72] Varrot A, Basheer SM, Imberty A. Fungal lectins: Structure, function and potential applications. Current Opinion in Structural Biology. 2013;23(5):678–85. 10.1016/j.sbi.2013.07.007 23920351

[73] Wang H, Ng TB, Ooi VEC. Lectins from mushrooms. Mycological Research. 1998;102(8):897–906. 10.1017/S0953756298006200

[74] Xu X, Yan H, Chen J, Zhang X. Bioactive proteins from mushrooms. Biotechnol Adv. 2011;29(6):667–74. 10.1016/j.biotechadv.2011.05.003 . 21605654

[75] Kawagishi H, Nomura A, Mizuno T, Kimura A, Chiba S. Isolation and characterization of a lectin from Grifola frondosa fruiting bodies. Biochim Biophys Acta. 1990;1034(3):247–52. Epub 1990/06/20. . 2364082 10.1016/0304-4165(90)90045-x

[76] Hassan MA, Rouf R, Tiralongo E, May TW, Tiralongo J. Mushroom lectins: Specificity, structure and bioactivity relevant to human disease. Int J Mol Sci. 2015;16(4):7802–38. Epub 2015/04/10. 10.3390/ijms16047802 25856678 PMC4425051

[77] Guillot J, Konska G. Lectins in higher fungi. Biochemical Systematics and Ecology. 1997;25(3):203–30. 10.1016/S0305-1978(96)00110-X

[78] Butschi A, Titz A, Walti MA, Olieric V, Paschinger K, Nobauer K, et al Caenorhabditis elegans N-glycan core beta-galactoside confers sensitivity towards nematotoxic fungal galectin CGL2. PLoS Pathog. 2010;6(1):e1000717 Epub 2010/01/12. 10.1371/journal.ppat.1000717 20062796 PMC2798750

[79] Ooi VEC, Liu F. A review of pharmacological activities of mushroom polysaccharides. International Journal of Medicinal Mushrooms. 1999;1(3):195–206. 10.1615/IntJMedMushrooms.v1.i3.10

[80] Wasser SP. Medicinal mushrooms as a source of antitumor and immunomodulating polysaccharides. Applied Microbiology and Biotechnology. 2002;60(3):258–74. 10.1007/s00253-002-1076-7 12436306

[81] Schimoler-O'Rourke R, Renault S, Mo W, Selitrennikoff CP. Neurospora crassa FKS protein binds to the (1,3)beta-glucan synthase substrate, UDP-glucose. Curr Microbiol. 2003;46(6):408–12. Epub 2003/05/07. 10.1007/s00284-002-3884-5 12732946. 12732946

[82] Kurita T, Noda Y, Yoda K. Action of multiple endoplasmic reticulum chaperon-like proteins is required for proper folding and polarized localization of Kre6 protein essential in yeast cell wall beta-1,6-glucan synthesis. J Biol Chem. 2012;287(21):17415–24. Epub 2012/03/27. 10.1074/jbc.M111.321018 22447934 PMC3366804

[83] Douglas CM. Fungal beta(1,3)-D-glucan synthesis. Med Mycol. 2001;39 Suppl 1:55–66. Epub 2002/01/22. . 11800269 10.1080/mmy.39.1.55.66

[84] Shahinian S, Bussey H. beta-1,6-Glucan synthesis in Saccharomyces cerevisiae. Mol Microbiol. 2000;35(3):477–89. Epub 2000/02/15. . 10672173 10.1046/j.1365-2958.2000.01713.x

