# Supplementary figures and images for: Transcriptome Analysis Revealed Highly Expressed Genes Encoding Secondary Metabolite Pathways and Small Cysteine-Rich Proteins in the Sclerotium of Lignosus rhinocerotis
Source: PLoS One. 2015 Nov 25;10(11):e0143549. doi: 10.1371/journal.pone.0143549 (PMC4659598; doi:10.1371/journal.pone.0143549)

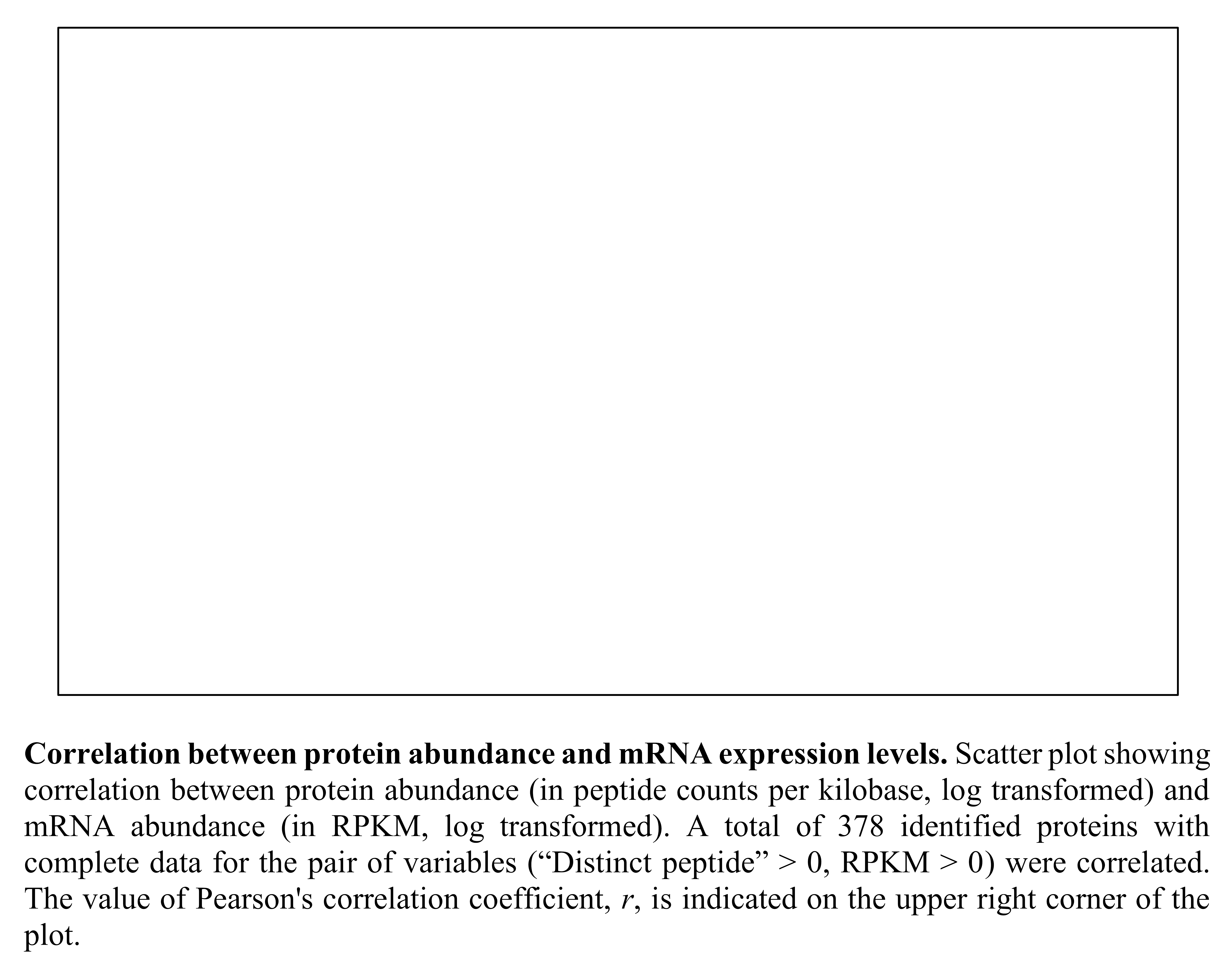

Supplement: S1 Fig — (TIF) [file pone.0143549.s001.tif]
